# Sub-wavelength extreme ultraviolet microscopy reveals domain-wall stability during ultrafast demagnetization

**DOI:** 10.1038/s41563-026-02583-w

**Published:** 2026-04-20

**Authors:** Hung-Tzu Chang, Sergey Zayko, Timo Schmidt, Ofer Kfir, Murat Sivis, Johan H. Mentink, Manfred Albrecht, Claus Ropers

**Affiliations:** 1 Department of Ultrafast Dynamics, Max Planck Institute for Multidisciplinary Sciences, Göttingen, Germany; 2 Institute of Physics, University of Augsburg, Augsburg, Germany; 3 School of Electrical & Computer Engineering, Tel Aviv University, Tel Aviv, Israel; 4 4th Physical Institute, University of Göttingen, Göttingen, Germany; 5 Radboud University, Institute for Molecules and Materials, Nijmegen, The Netherlands

**Keywords:** Magnetic properties and materials, Ultrafast photonics, Imaging techniques, Surfaces, interfaces and thin films

## Abstract

Ultrafast control of magnetic textures relies on understanding how domain walls respond to femtosecond laser excitation. Previous X-ray scattering studies suggested transient domain-wall broadening and motion during reversible demagnetization. Yet such dynamics have never been observed directly, owing to insufficient spatiotemporal resolution in existing techniques. Here we introduce femtosecond extreme ultraviolet microscopy with sub-wavelength 13.5-nm spatial resolution, enabling direct real-space imaging of domains in ferri- and ferromagnetic thin films during laser-induced demagnetization. Within an experimental precision better than 2 nm, domain-wall positions and widths remain unchanged up to the onset of irreversible magnetic switching at 50%–80% demagnetization. These findings establish quantitative limits for reversible domain-wall motion and spin transport upon ultrafast demagnetization, while implying that irreversible reconfiguration underlies characteristic changes in diffraction signatures at high fluence. The approach expands time-resolved microscopy into the nanometre and femtosecond regime, enabling element-specific studies of coupled spin, charge and lattice dynamics in quantum and functional materials.

## Abstract (web summary)

Femtosecond extreme ultraviolet microscopy is used to directly image domain-wall dynamics during laser-induced demagnetization. The domain walls are found to be stable up to the onset of irreversible magnetic switching.

## Main

Photoinduced demagnetization^1,2^ underlies the ultrafast manipulation of spins, which can drastically accelerate data processing and facilitate the development of next-generation information storage and spintronics^3–5^. Over the past decades, the mechanisms of ultrafast demagnetization in homogeneously magnetized samples, including energy transfer to the spin system^1^, loss of angular momentum due to spin-flip scattering and phonon excitation^6–8^, as well as non-local spin transport^9,10^, have been proposed, observed and explored in great detail. On the other hand, photoinduced real-space dynamics of nanoscale spin textures have largely remained elusive, due to the lack of tools combining nanometre spatial and femtosecond temporal resolution. Even for magnetic domain walls (DWs)—the quintessential element of magnetic textures and integral parts of electronics and information storage devices—existing approaches have not yet provided a unified and definitive picture of their dynamics after photoexcitation. Contemporary studies on ultrafast demagnetization in the presence of magnetic domains mainly use time-resolved extreme ultraviolet (XUV) or soft X-ray scattering in reciprocal space to infer dynamics at the nanoscale. These experiments have reported a large variety of DW dynamics, including domain fluctuations^11^, DW broadenings up to several tens of nanometres or ~50% of their original widths^12–14^, as well as DW motions with velocities reaching ~70 km s^−1^ (refs. ^13–17^), far exceeding the Walker breakdown velocities at which the DW structure is expected to become unstable^18^. Those dynamics were usually attributed to non-local superdiffusive spin transport^12,13,19^ or a transient decrease in magnetic anisotropy^14^. In addition, many of the reported phenomena exhibit highly nonlinear fluence dependence^12,20^ and threshold-like behaviour^17^. Although the diverse set of experimental observations illustrates the richness of non-equilibrium magnetic phenomena, it also demonstrates the challenges involved in developing a direct and comprehensive approach to inspect and elucidate processes in ultrafast magnetism. Scattering experiments in reciprocal space enable the extraction of nanoscale statistical properties of domains and DWs. However, this inevitably relies on assumptions on the domain morphology and DW profiles, as discussed in, for example, refs. ^11,13^. Here we utilize ultrafast XUV sub-wavelength imaging to provide a direct real-space view on nanoscale DWs upon photoexcitation.

**Fig. 1:**
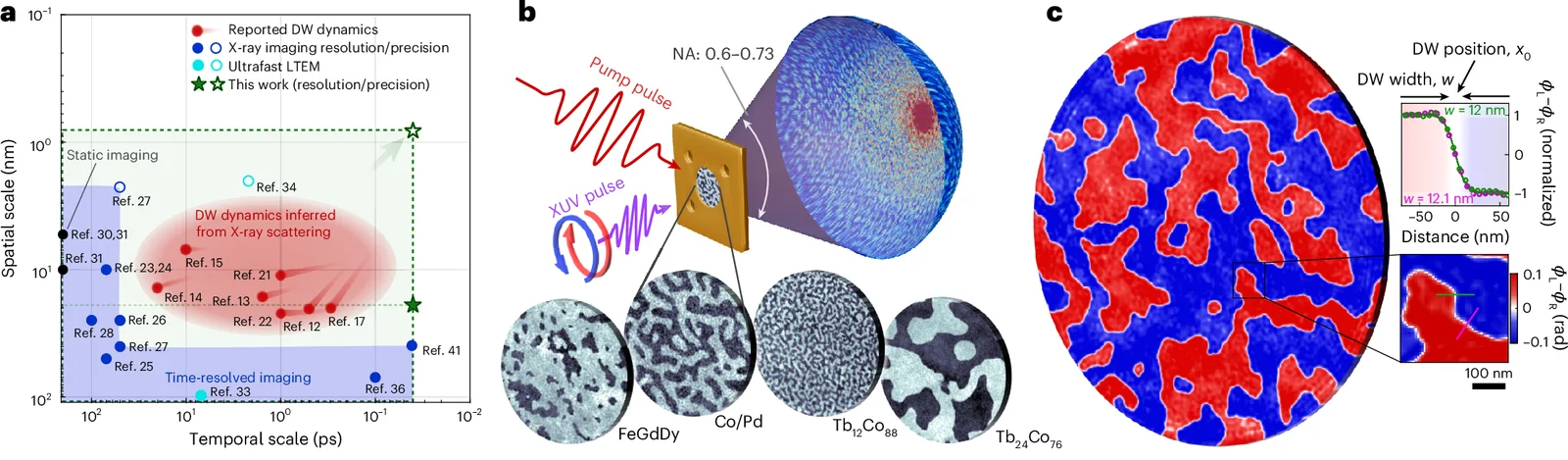
Ultrafast sub-wavelength imaging of nanoscale spin textures using a tabletop HHG source. **a**, Literature overview highlighting current magnetic imaging capabilities. The spatial resolution and precision are labelled with filled and open circles, respectively. Blue circles represent time-resolved X-ray magnetic imaging, and the results obtained with Lorentz transmission electron microscopy (LTEM) are shown in cyan. The estimated DW dynamics inferred from X-ray scattering experiments are indicated as red dots, with tails illustrating the reported temporal evolution. The spatiotemporal resolution and DW measurement precision achieved in this work are represented by green stars. **b**, Schematic of the experimental setup for optical–XUV pump–probe magnetic imaging. The sample, partially covered by an opaque mask defining a circular FOV, is uniformly excited from the magnetic film side by femtosecond laser pulses. Circularly polarized high-harmonic pulses resonantly probe the magnetic contrast at the corresponding absorption edges (53 eV for Fe-based and 60 eV for Co-based samples), capturing the induced magnetization dynamics in real space via lensless imaging. The diameter of the FOV is 3 μm. NA, numerical aperture. **c**, Example static image of the magnetic domain pattern in Tb_26_Co_74_ alloy, recorded with 13.5-nm spatial resolution using a wavelength of 20.8 nm. Right: magnified region and two line profiles taken along the marked lines. Fitting with equation (1) returns the local DW width parameter *w* and position *x*_0_ of the corresponding DW.

Photoinduced magnetization dynamics typically unfold on sub-picosecond temporal and sub-20-nm spatial scales. Figure 1a summarizes the literature on DW dynamics inferred from XUV and X-ray scattering studies (red-shaded region and reported changes in DW width)^12–17,21,22^. Time-resolved magnetic imaging experiments can, thus far, not reach such scales simultaneously (Fig. 1a, blue-shaded region). Specifically, soft X-ray microscopy at synchrotrons has achieved spatial resolution down to 10 nm (refs. ^23–28^), with recent advancements extending static magnetic imaging to 5 nm (refs. ^29–31^). Although this is sufficient to map typical DW profiles^29^, the corresponding temporal resolution of soft X-ray imaging with synchrotron sources is currently limited to tens of picoseconds^32^. Cutting-edge transmission electron and scanning tunnelling microscopy enable the tracking and mapping of spin textures with a spatial precision down to 2 nm and 0.4 nm, respectively^33–38^, but access to femtosecond spin dynamics at such scales has not yet been reported. Time-resolved magnetic imaging with free electron lasers offers femtosecond resolution at spatial resolution down to about 70 nm (ref. ^39^). Tabletop high-harmonic generation (HHG) sources feature exceptional time resolution and comprehensive polarization control in the XUV range^40–42^, with which, nevertheless, it is still challenging to reach the spatial scales characteristic of DWs. In particular, common ferromagnetic materials such as Ni, Co and Fe feature absorption edges between 18 and 24 nm, where magnetization-sensitive contrast can be obtained through X-ray magnetic circular dichroism (XMCD). Resolving DW properties at these wavelengths thus requires imaging with a resolution at or below the wavelength.

In this work, we track the local properties of nanoscale DWs during ultrafast demagnetization using time-resolved imaging with a femtosecond XUV source, and reveal unexpected DW stability after intense laser irradiation. Reaching 13.5-nm sub-wavelength spatial and under-40-fs temporal resolution, we map the spatial distributions of magnetic domain boundaries in ferrimagnetic TbCo and ferromagnetic Co/Pd thin films. These material systems were chosen because prior far-field scattering experiments had reported pronounced DW dynamics during ultrafast demagnetization, rendering them prototypical systems to study using direct real-space imaging. In addition, some TbCo sample compositions close to the angular-momentum compensation point were selected to maximize DW mobility^43^ and thus provide the most favourable conditions to detect DW motion.

Using detailed quantitative image analysis, we achieve a spatial precision below 2 nm in determining DW widths and positions, and rule out transient DW broadenings or displacements for demagnetization levels up to approximately 60%. The width of the spatially averaged DW profiles (*w*_avg_) measured in each individual time frame as well as the average local DW width ($\bar{w}$) remains invariant within ~1 nm across all time frames (1*σ* < 0.2 nm). At higher pump fluences inducing even stronger demagnetization levels, stochastic irreversible domain switching becomes prominent. Our results present a direct and spatially resolved picture of local spin dynamics during laser-induced demagnetization, and establish upper bounds for reversible ultrafast DW dynamics caused by, for example, spin transport.

## Results

Our experiments use an optical-pump, XUV-probe scheme, which is depicted in Fig. 1b and detailed in Methods and Supplementary Information. In brief, the sample is excited with femtosecond laser pulses centred at around 800 nm and stroboscopically probed by time-delayed, circularly polarized XUV pulses at the corresponding core-level absorption edge. For example, the probe wavelength for Co-based samples is 20.8 nm (Co M_2,3_ edge, ~60-eV photon energy). An opaque mask with a 3-μm-diameter circular field of view (FOV) is placed in close proximity to the magnetic film to confine the XUV illumination. The scattering signal is recorded in the far field, and the amplitude and phase of the electric field exiting the sample are numerically reconstructed using iterative phase retrieval. For each magnetic image, two sets of data are recorded with left- and right-circularly polarized XUV pulses, respectively, to isolate the magnetic signal from non-magnetic contribution via XMCD. The illumination wavelength is tuned to maximize the phase difference contrast (*ϕ*_L_(**r**) − *ϕ*_R_(**r**)) at the core-level absorption edge. Here **r** denotes the position vector within the FOV. Example domain patterns in different materials and compositions are shown in Fig. 1b,c. In particular, we show domain images of both Fe- and Co-based samples (Fig. 1b), where the magnetic contrasts originate from Fe and Co M_2,3_ edges, respectively, which demonstrates the versatility of the imaging approach across different materials and compositions. Note that Fig. 1c presents an image of a ferrimagnetic sample close to its magnetic compensation point, where opposing magnetic sub-lattices of Co and Tb cancel the net magnetization. Owing to the element-specific contrast of the imaging scheme, the domain texture is clearly resolved. A consistent isotropic sub-wavelength resolution of 13.5 nm is achieved, with maximum scattering angles corresponding to a numerical aperture of 0.73. Further details on the quantification of spatial resolution based on Fourier ring correlation, phase retrieval transfer function and real-space analysis are provided in Supplementary Information.

Real-space images enable the direct extraction of local DW profiles (Fig. 1c, right), where two example lineouts perpendicular to a DW are taken. For thin films with strong perpendicular magnetic anisotropy, DWs can be described by a hyperbolic tangent function^13,14,44,45^:

1 $${M}_{z}(x)={M}_{0}\tanh \frac{x-{x}_{0}}{w},$$

where *x* denotes the position along a lineout perpendicular to the DW contour, *M*_0_ is the out-of-plane magnetization of the magnetic domain, *x*_0_ is the centre position of the DW and *w* is its width parameter. To obtain local DW profiles throughout the FOV, we first identify a coarse set of DW locations using contours with *ϕ*_L_(**r**) − *ϕ*_R_(**r**) = 0. Coordinates for the profiles {*x*} are, thus, lines perpendicular to the contours at every point. To ensure unbiased measurements, the contours used to determine DW locations are identified first and kept fixed for DW width measurements in subsequent images. At each location along the contour, the local width parameter *w* and the centre position *x*_0_ are obtained by fitting equation (1) to the isolated DW profile.

**Fig. 2:**
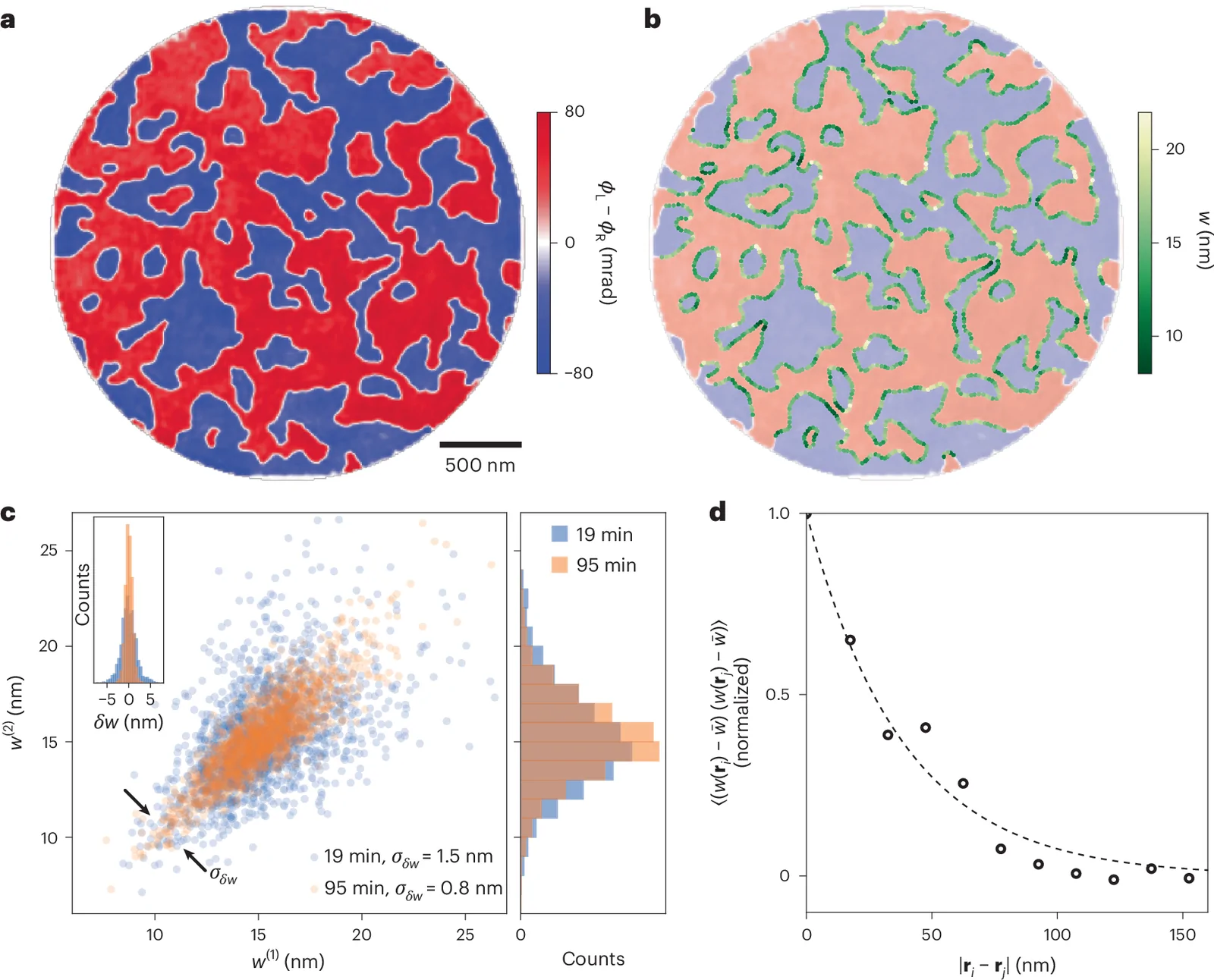
Magnetic domain imaging before optical excitation (time delay *t* < 0). **a**, Example of a magnetic domain texture in a Tb_25_Co_75_ alloy sample. **b**, Local DW width across the FOV is shown in a green–yellow colour map. The fitted DW width is 15.2 ± 2.1 nm. **c**, Correlation plots of the DW widths extracted from 2 independent measurements using a total of ~19 min (blue circles) and ~95 min (orange circles) of exposure time per image. The spread of the off-diagonal distribution (inset) represents the difference in DW widths measured at the same locations in the 2 independent datasets, indicating the measurement precision (standard deviation *σ*_*δw*_ reaching 0.8 nm). **d**, Correlation function of the DW width with respect to the distance between DW locations (circles), which is fitted with a single exponential function $\exp (-| {{{\mathbf{r}}}_{i}}-{{{\mathbf{r}}}_{j}}| /{D}_{w})$ (dashed line) with decay length *D*_*w*_ = 39 nm.

Figure 2a,b shows an example of DW mapping in a Tb_25_Co_75_ sample. The local DW profiles are first identified across the FOV, and their extracted widths are shown in Fig. 2b using a green–yellow colour map. The measured DW width in this sample follows a normal distribution, with an average of $\bar{w}=15\;{{\rm{nm}}}$ and a standard deviation *σ*_*w*_ = 2.1 nm. It is important to note that at a given spatial resolution, the precision of extracting the local DW properties is limited by the signal-to-noise ratio of the image, which is governed by the exposure time. To determine the precision of the local DW width measurement, we compare the extracted DW widths of the same FOV (Fig. 2a) from two independently measured and separately reconstructed datasets. Figure 2c shows the correlation of the DW widths from the two datasets denoted as *w*^(1)^ and *w*^(2)^, respectively. Here each dot represents the local DW width at the same location in the two datasets. The standard deviation of the histogram *σ*_*δw*_ of the data points along the off-diagonal direction (Fig. 2c, inset) indicates the degree of consistency between the two measurements. For the dichroic images acquired with a total of 1,136 s of exposure time (blue circles), *σ*_*δw*_ is 1.5 nm and it diminishes to 0.8 nm when the exposure time is increased by a factor of 5 (orange circles). Meanwhile, the distribution of DW widths across the FOV (Fig. 2c, right) also narrows from 2.4 nm to 2.1 nm. As the standard deviation of the DW width distribution is larger than the measurement precision and the decrease in *σ*_*w*_ is small compared with the increase in precision, the distribution of DW widths represents the intrinsic variation of DW profiles across the sample.

In Fig. 2b, the characteristic changes in DW width along the contours occur on length scales substantially larger than the spatial resolution, suggesting contributions from local sample properties and domain topology. In addition, *w* tends to shrink when there is another DW in close proximity. Similar correlations between DW width and domain size have been observed in two-dimensional van der Waals magnets^46^, and the variation in local DW properties was theoretically explored with the application of exchange bias^47^. For the TbCo sample, the phenomenon can be explained by the inhomogeneity of atomic compositions. Such inhomogeneity has also been observed in ferrimagnets such as GdFeCo (ref. ^48^). The difference in atomic compositions leads to variations in the strength of exchange interaction across the sample. When the exchange interaction is reduced, smaller domain sizes become more favourable, and the DW narrows^44^. To assess the inhomogeneity of DW widths, we plot the correlation function with respect to the distance between wall locations (Fig. 2d, black circles), which is defined as $\langle (w({{\mathbf{r}}}_{i})-\bar{w})(w({{\mathbf{r}}}_{j})-\bar{w})\rangle /\langle {(w({{\mathbf{r}}}_{i})-\bar{w})}^{2}\rangle$, with **r**_*i*_ denoting the *i*th DW location. The width correlation function can be modelled by a single exponential (dashed line) with a decay length of 39 nm and, thus, can serve as a measure of sample inhomogeneity.

**Fig. 3:**
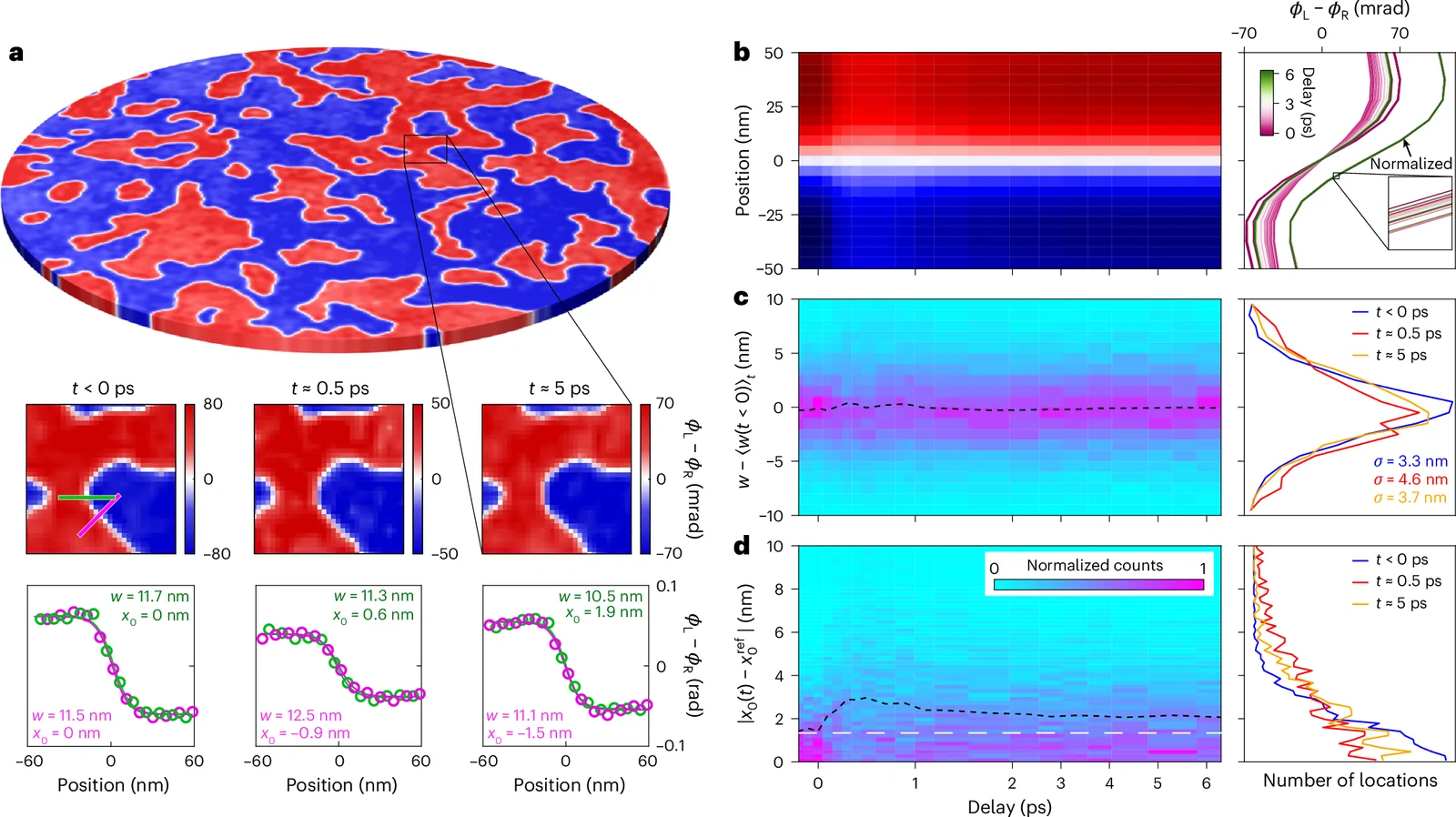
Ultrafast imaging during demagnetization at a pump fluence of 5.5 mJ cm^−2^, inducing ~37% demagnetization. **a**, Magnetic domain pattern in the Tb_25_Co_75_ sample before optical excitation (*t* < 0). The magnified region shown in **a** highlights the magnetic state before optical excitation (*t* < 0 ps), close to maximum demagnetization (*t* ≈ 0.5 ps) and during recovery (*t* ≈ 5 ps). Bottom: time evolution of an example DW during demagnetization. The locations of the corresponding DWs are shown with green and magenta lines on the image taken at a negative delay. **b**, Averaged DW profiles across the FOV as a function of time delay. Right: averaged DW profiles at different delay times. The curves on the right are the same curves normalized to the negative delay. **c**, Histograms of extracted width parameters $w({{\mathbf{r}}},t)$ for all DW locations across the FOV compared with the corresponding width at negative delays (${\langle w({{\mathbf{r}}},t < 0)\rangle }_{t}$). Right: three example histograms corresponding to the delays marked in **a**. **d**, Time-dependent distribution of the magnitudes of displacements. The averaged magnitudes of displacements (black dashed line) exhibit a baseline of 1.5 nm (white dashed line), representing the precision in repeated measurements of individual DWs.

Having established the imaging resolution and precision in determining DW properties, we next discuss the behaviour upon photoexcitation. Figure 3a illustrates the time-dependent magnetization dynamics of Tb_25_Co_75_ at three characteristic delays: the unpumped state (*t* < 0 ps), near the maximum magnetization suppression (*t* ≈ 0.5 ps) and during relaxation (*t* ≈ 5 ps). Throughout the FOV, we observe a decrease in overall magnetization within 0.4 ps, reaching *M*_*z*_(0.4 ps) ≈ 0.63*M*_*z*_(*t* < 0 ps), and a partial recovery after a few picoseconds. In this time-resolved dataset, we first analyse the temporal evolution of the spatially averaged DW profiles (Fig. 3b) and the resulting width *w*_avg_(*t*). The time-dependent profiles become indistinguishable from each other when normalized (Fig. 3b, right), and the statistical average of *w*_avg_(*t*) for both positive and negative delays is 15.1 ± 0.2 nm, indicating that there is no statistically significant increase in the DW width after optical excitation. In addition, the standard deviation at negative times (*σ*(*w*_avg_(*t* < 0)) = 0.2 nm) reflects the high precision in measuring *w*_avg_.

To systematically characterize the potential changes in local DW widths *w*, the histograms of the time-dependent difference $\Delta w=w({{\mathbf{r}}},t)-{\langle w({{\mathbf{r}}},t < 0)\rangle }_{t}$, representing the deviation of *w*(*t*) from its unpumped value *w*(*t* < 0), are displayed in Fig. 3c. The corresponding time-dependent average of local changes in DW widths (${\langle \Delta w({{\mathbf{r}}},t)\rangle }_{{\mathbf{r}}}$) is plotted as a black dashed line (Fig. 3c, left). The distribution of ${\langle \Delta w({{\mathbf{r}}},t)\rangle }_{{\mathbf{r}}}$ exhibits an average and standard deviation of 0.0 ± 0.2 nm, consistent with the results from the averaged DW profiles (Fig. 3b). Figure 3c (right) shows the representative histograms of Δ*w*(*t*) at three different time delays. If DW broadening occurs at some locations, the time-dependent distribution of Δ*w* is expected to skew towards positive values for *t* > 0 ps. Compared with negative delays (blue line), the distributions at positive delays retain the symmetric shape, with the maxima centred at around Δ*w* = 0, providing no indication of a systematic increase in DW width. The observed change in the distribution width in Δ*w* by ~1.3 nm as magnetization is suppressed (red line) is attributed to the diminishing XMCD contrast in the demagnetized state. This reduction in contrast effectively lowers the signal-to-noise ratio of the magnetic image, leading to a somewhat greater uncertainty in the extraction of DW properties (Supplementary Fig. 4).

Using an analogous approach to the width analysis, we now investigate potential DW displacements within the same dataset. The magnitude of displacements, $| {x}_{0}(t)-{x}_{0}^{{\rm{ref}}}|$, relative to their reference positions at negative delays, are computed for each location (${x}_{0}^{{\rm{ref}}}={\langle {x}_{0}(t < 0)\rangle }_{t}$). Figure 3d displays their distributions across the FOV at different delays. The average displacement measured at *t* < 0, is approximately 1.5 nm, representing the baseline uncertainty in determining the DW positions (white dashed line). The average of the absolute displacement as a function of time ($\langle | {x}_{0}(t)-{x}_{0}^{{\rm{ref}}}| \rangle$, black dashed line) increases to approximately 3 nm when the sample undergoes demagnetization and subsequently decays as the magnetization recovers. Figure 3d (right) displays the histograms of $| {x}_{0}(t)-{x}_{0}^{{\rm{ref}}}|$ at three representative delays. All the distributions peak at zero displacement and broaden at positive delays. However, this broadening can be attributed solely to the increased fitting uncertainty caused by the reduced XMCD contrast. This assignment is supported by Monte Carlo simulations using artificial DW profiles (Supplementary Text 3 and Supplementary Fig. 5). Furthermore, binning experimental data from adjacent delays narrows the width of the distributions and lowers the magnitude of the displacements by about 50% (Supplementary Fig. 6). Therefore, the averaged magnitude of DW displacement must be less than 3 nm and is likely to be considerably smaller.

**Fig. 4:**
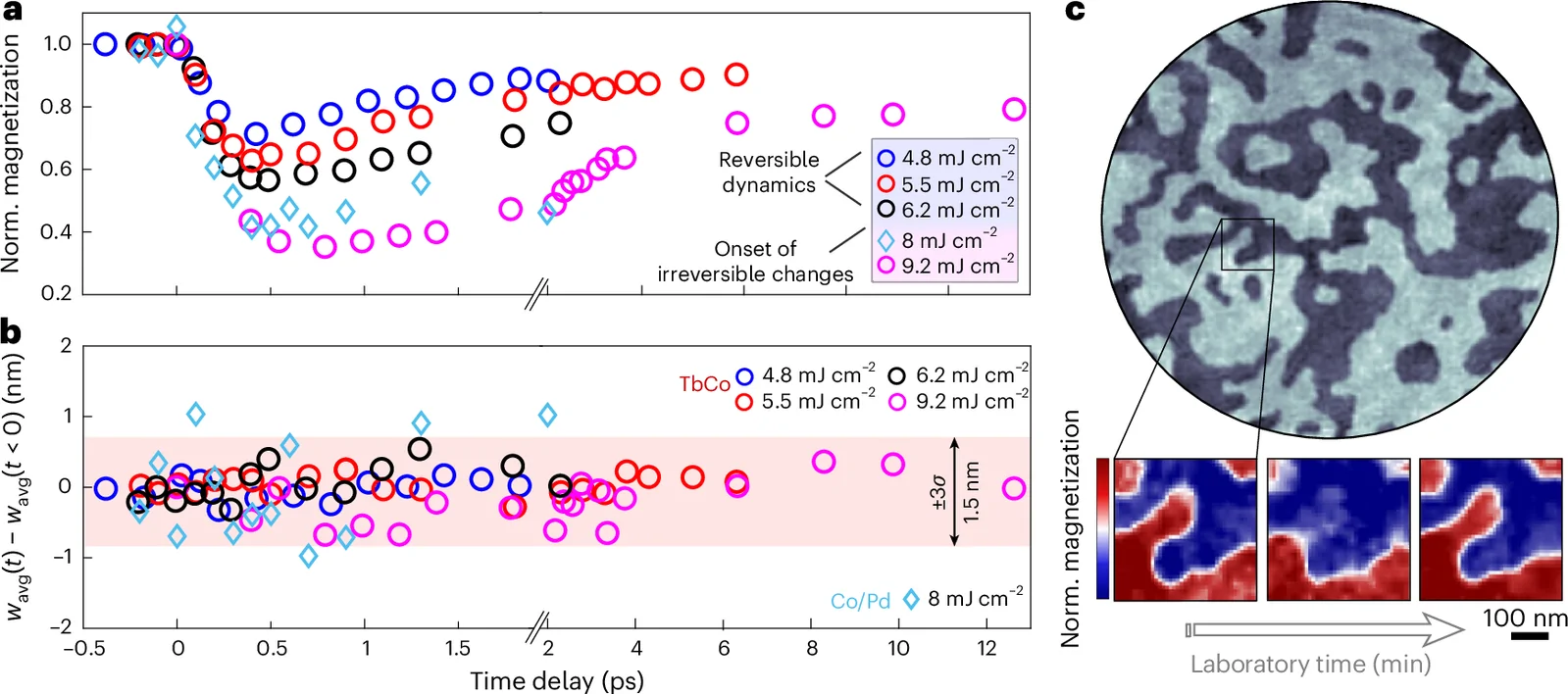
DW stability at various pump fluences. **a**, Spatially averaged magnetization (∣*M*_*z*_∣) across the FOV, normalized (norm.) to the values at negative delays. **b**, Time-dependent changes in widths of the spatially averaged DW profiles compared with negative delays (${w}_{\mathrm{avg}}(t)-{\langle {w}_{\mathrm{avg}}(t < 0)\rangle }_{t}$) for each pump fluence shown in **a**. The combined distribution of ${w}_{\mathrm{avg}}(t)-{\langle {w}_{\mathrm{avg}}(t < 0)\rangle }_{t}$ for all measured fluences with TbCo exhibits a standard deviation of *σ* = 0.25 nm, and the corresponding *σ* for Co/Pd is 0.7 nm. The red-shaded area indicates the ±3*σ* interval of the combined dataset recorded using the TbCo sample. **c**, Example of delay-independent, local stochastic domain switching in Tb_25_Co_75_ at a pump fluence of 9.2 mJ cm^−2^. The 3 images were acquired consecutively, with an exposure time over several minutes each, that is, >10^5^ pump–probe laser shots.

We further investigate the potential DW dynamics at different pump fluences in the Tb_25_Co_75_ sample, focusing on *w*_avg_. The time-dependent magnetization and change in *w*_avg_ with respect to its unpumped value (${\langle {w}_{\mathrm{avg}}(t < 0)\rangle }_{t}$) under different pump fluences are displayed in Fig. 4a,b, respectively. In particular, *w*_avg_(*t*) remains largely unchanged irrespective of fluence. For Tb_25_Co_75_, the distribution of ${w}_{\mathrm{avg}}(t)-{\langle {w}_{\mathrm{avg}}(t < 0)\rangle }_{t}$ presents an average and standard deviation of 0.0 ± 0.3 nm under pump fluences of up to 9.2 mJ cm^−2^. The Co/Pd sample exhibits similar behaviour, though with a moderately larger standard deviation of 0.7 nm due to a lower XMCD contrast. Since both DW broadening and displacement would result in an increase in *w*_avg_, its invariant width suggests that the overall domain structures remain stable for magnetization suppression down to approximately 40% (*M*_*z*_ ≈ 0.4*M*_*z*_(*t* < 0); Fig. 4a,b).

At high pump fluences inducing demagnetization above approximately 50%, irreversible changes in the magnetic domain patterns emerge in certain regions of the samples. Figure 4c illustrates the onset of these changes at a fluence of ~9 mJ cm^−2^ in Tb_25_Co_75_. In the magnified region, magnetization switching is observed. Note that for the aforementioned results of DW widths and displacements (Fig. 4a,b), regions with irreversible changes are first identified and omitted during data processing. In our stroboscopic experimental scheme, the reconstructed image corresponds to the average magnetic configuration measured over many pump–probe cycles (Supplementary Text 4). When stochastic switching is infrequent compared with the number of measurement cycles for each image, such dynamics are reflected by abrupt changes in the XMCD contrast of successive images (Fig. 4c). At higher pump fluences, irreversible dynamics occur more frequently, at more locations and with larger areas (Supplementary Figs. 8 and 9). With increasing switching frequency, the magnitude of the average XMCD contrast during the acquisition period diminishes towards zero, resulting in a weaker contrast compared with the non-switched areas.

## Discussion

The discovered stability of DWs calls for a reconsideration of the frequently observed ultrafast changes in X-ray diffraction from magnetic thin films. Specifically, our results on two prototypical magnetic systems demonstrate that reversible demagnetization is not associated with transient changes to DW widths and locations beyond the scale of 2 nm (Fig. 4b).

The observation of stochastic switching calls for future studies to take irreversible dynamics into account. Many of the X-ray and XUV scattering studies reporting changes to scattering angles were conducted at high pump fluences^12,13,17^. The direct observation of stochastic switching in our data strongly suggests that such changes to the scattering signal may involve or coincide with irreversible changes in the domain structure, particularly in regions where the domain arrangement becomes highly unstable. In addition, our data at high pump fluences indicate that regions consisting of small domains are more prone to local switching (Supplementary Fig. 9), which may result in intensity suppression at high diffraction angles and an effective shrinkage of diffraction rings in scattering. The observed upper bounds for possible DW broadenings agree with the nanometre spin propagation lengths measured in spintronic emitters^49^ and the estimation for the loss of magnetic anisotropy from quasi-thermal heating (Supplementary Text 5). Furthermore, as our measurement window with delays up to >40 ps (Fig. 4 and Supplementary Fig. 10) spans the characteristic timescales for superdiffusive transport as well as spin and phonon heating^1,9^, the observed upper bounds apply to both transport- and heat-driven reversible DW dynamics under homogeneous laser excitation.

The fluence dependence of the dynamics observed in several scattering experiments bears similarity to our observations^12,17,20^. For example, at a fluence inducing a maximum level of ~20% demagnetization, no DW dynamics can be inferred from time-resolved scattering experiments in Co/Pd multilayers^20^, whereas DW broadenings of ~50% were derived for a demagnetization amplitude of ~70% (ref. ^12^). Although such a difference was attributed to the nonlinear fluence dependence and the differences in spatial distribution of optical excitation^20^, our results provide an alternative view. At low to moderate fluence, the DWs remain inert, whereas at high fluence, stochastic switching emerges, leading to a change in the diffraction signal. The behaviour of irreversible switching can be further connected to recent reports on all-optical switching^50–53^, where the domain nucleation also exhibits a fluence threshold^51,53^. The threshold behaviour and stochastic nature of the observed switching suggests a thermal-activation-type mechanism^53^: after laser excitation, the spins in the sample are heated, and smaller domains with higher energies, that is, a larger ratio between the length of the DW and the domain area, are easier to overcome the energy barrier and switch. The relative instability of small domains may serve as a precursor for full optical switching. In addition, it may indicate the existence of a transient state during demagnetization in which local magnetic moments rapidly fluctuate before stabilizing into a new configuration^11,54^.

Our work, in conjunction with studies on magneto-optical switching^50–53^ and skyrmion nucleation^55–58^, suggest that thermal fluctuations play a major role in optical processes involving magnetic domains. By contrast, the presented results indicate a limited influence of spin transport. The observed upper limit of DW motion and the implications of contributions from different mechanisms to domain dynamics are relevant for the development of future magnetic memory devices. In particular, our results identify conditions under which femtosecond laser pulses can be used to transiently soften magnetic structures without inducing unintended DW motion. The exceptional robustness of DWs also implies the feasibility of developing ultrafast and ultrasmall all-optical magnetic toggle^59^.

The demonstrated sub-wavelength full-field XUV microscope provides a powerful platform for exploring ultrafast magnetism at its fundamental length scales and timescales. The core-level XUV probe is element specific, enabling access to various magnetic systems including ferro- and ferrimagnets, and magnetic semiconductors and insulators. It can interrogate complex systems even near the magnetic compensation point, as shown for TbCo, and facilitates studying compensated and altermagnetic materials^60^. Beyond magnetism, the method offers real-space insights into nanoscale chemical and structural dynamics^61–64^, and even holds promise for high-resolution metrology in the context of XUV lithography. This unique combination of spatiotemporal precision, material sensitivity and broad applicability renders XUV microscopy a highly promising tool for future ultrafast materials science.

## Methods

### Sample preparation

The ferromagnetic sample used is a Co/Pd multilayer with the following structure: Co (0.5 nm)/Pd (0.75 nm) repeated nine times. The ferrimagnetic films are Tb_1−*x*_Co_*x*_ alloys with a thickness of 20 nm. For all datasets except those shown in Fig. 1b, *x* = 0.74 or 0.75, and for those in Fig. 1b, *x* varies between 0.74 and 0.88. All the samples exhibit strong perpendicular magnetic anisotropy with easy-axis magnetization pointing out of plane. The samples were prepared at room temperature by d.c. magnetron sputtering from elemental targets onto 200-nm-thick silicon membranes. The sputtering process was carried out with argon gas at a working pressure of 3.5 × 10^−3^ mbar in a vacuum chamber (base pressure, <10^−8^ mbar). To protect the films from oxidation, 2 nm of Pd or 5 nm of Si_3_N_4_ was used as a cover layer. The layer thicknesses were estimated using areal densities measured by a calibrated quartz balance before deposition.

For the imaging mask, 200 nm of gold was deposited on the backside of the samples via thermal evaporation. At locations separated by >300 μm, the gold film was selectively etched with a focused ion beam to create circular apertures with a 3-μm diameter as the FOV. Several auxiliary holes, with diameters ranging from 200 to 600 nm, were milled through the entire structure to facilitate scattering data collection at a high numerical aperture. On some masks, two additional reference holes (with diameters of 200 and 300 nm) were fabricated to serve as reference sources for Fourier transform holography. The holographic reconstruction is used only to provide the initial guess for the real-space support in the phase retrieval process. Compared with the mask design in ref. ^41^, the arrangement of auxiliary holes is optimized to meet increased longitudinal coherence requirements by reducing the total size along the axis of spectral dispersion (Supplementary Fig. 7).

### Experimental setup

The optical–XUV pump–probe setup is driven by a Ti:sapphire laser with wavelength centred at around 800 nm. The laser is operated at a 1-kHz repetition rate with a pulse duration of 33 fs. The laser output is split into a pump and a probe arm with a 1:9 beamsplitter. The pump arm is time delayed with the probe arm by a motorized delay stage and the pulse energy is fine-tuned with neutral density filters. The probe arm encompasses an in-line, bichromatic, circularly polarized HHG setup using helium as the generation medium. The generated XUV light is first separated with the fundamental driving beam with a 200-nm-thick Al filter and subsequently dispersed and wavelength selected by a set of toroidal grating and a slit. After the slit, the quasi-monochromatic XUV beam illuminates the sample and the transmission is captured by a charge-coupled device camera. To achieve the resolution and imaging precision, the stability of the overall system, beam geometry and coherence of the XUV light are carefully optimized through the design of the setup (Supplementary Text 1).

### Data collection and iterative phase retrieval

The diffraction data are collected using 1,024 × 1,024 pixel^2^ for time-resolved data and 2,048 × 2,048 pixel^2^ for static high-resolution images (Fig. 1b,c). Dark images without XUV exposure were taken and subtracted from the diffraction images. To account for the high dynamic range of the scattering signal, several acquisitions with varying exposure times are recorded and merged. Short exposure times capture only low-frequency components, typically ranging from 0.5 to 5 s, depending on the numerical aperture used, whereas longer exposure times are typically between 20 and 60 s. For example, to obtain a single real-space image of the time series shown in Fig. 3a, the average of eight acquisitions at 0.5 s each was combined with an averaged image of ten acquisitions at 28 s each to form a single high-dynamic-range diffraction pattern. This approach is sufficient to achieve high-quality real-space image with sub-wavelength resolution for a single helicity. Nevertheless, the magnetic signal in such an image is weak and overwhelmed by non-magnetic contributions. To isolate the magnetic signal, an identical dataset with opposite circular polarization is acquired. Therefore, the total exposure time required for a single time-delay image in Fig. 3 is (0.5 s × 8 + 28 s × 10) × 2 = 568 s. Longer exposure times improve the image quality and, thus, the precision of DW width and position. Before conducting iterative phase retrieval, the diffraction data are projected onto the Ewald sphere to account for curvature at high scattering angles^65^.

The autocorrelation from the Fourier transform of the diffraction image is used to create the imaging support for iterative phase retrieval. Due to the presence of distant reference holes (Supplementary Fig. 7), the autocorrelation contains cross-correlation terms between the mask and the reference holes. This cross-correlation (Fourier transform holography reconstruction of the object) provides the shape of the mask, with resolution governed by the size of the smallest reference hole. The real-space pixel size of this reconstruction is inversely related to the spatial frequency at the edge of the detector *q*, given by pixel size = 1/(2*q*). Since the distance between the reference holes is known from the focused-ion-beam design, the spatial frequency at the edge of the detector can be calibrated without exact knowledge of the sample-to-detector distance.

The iterative phase retrieval process begins with data recorded under left-circularly polarized light and a random initial guess. First, we run 100 iterations of the relaxed averaged alternating reflections algorithm^66^, with a relaxation parameter of 0.9. In the following 300 iterations, the relaxation parameter is gradually reduced from 0.9 to 0.5. An additional 20 iterations of error reduction^67^ are then applied to finalize the preliminary reconstruction of left-circularly polarized light.

After this initial run, the real-space support is updated via thresholding the real-space image. The entire process is then repeated three more times to improve the accuracy of real-space support. The final support is subsequently used for reconstructing all data recorded with this mask and numerical aperture, for example, all frames within one time-resolved experiment.

The preliminary reconstruction process assumes monochromatic, perfectly coherent light and accounting for partial coherence due to imperfections in the optical system is required to lower the reconstruction error. To achieve this, an additional 200 relaxed averaged alternating reflection iterations are performed, with the mutual coherence function estimated every 10th iteration using 15 steps of Richardson–Lucy deconvolution^29,68^. The final reconstruction then serves as the initial guess for reconstructing the data recorded with the opposite helicity, for which only 20 iterations of error reduction are required.

The phase difference of the reconstructions recorded with opposite helicities forms the phase-contrast image (*ϕ*_L_ − *ϕ*_R_) (Figs. 1–4), which is proportional to the out-of-plane magnetization of the sample^40^. Although the first image can serve as an initial guess for subsequent reconstructions, for example, in a time-dependent measurement, such practice is avoided to prevent potential bias towards a close or similar solution. In this work, all time-resolved datasets are reconstructed independently using random initial guesses, with no overlap between the data recorded at different times.

It is important to note that the real-space support determines the exact plane of the reconstructed exit surface wave. When the support is loose, the final reconstruction may appear blurred or defocused and must be numerically propagated to the correct plane at the exit surface. With a well-defined, tight support, the reconstruction algorithm reliably converges to an effectively identical solution across the momentum space, regardless of the initial guess. The convergence is verified by the phase retrieval transfer function and Fourier shell correlation analysis (Supplementary Fig. 1), where both metrics exceed the threshold criteria across the entire momentum space, indicating isotropic diffraction-limited resolution down to 13.5 nm.

### Fitting of DW profiles

Before the extraction DW properties, a super-Gaussian filter is applied to smooth the edges of the far-field data (Supplementary Information). The resulting magnetic image is then interpolated and mapped onto a grid with spacing equal to half the real-space pixel size. The positions of DWs are localized using contours with *ϕ*_L_(**r**) − *ϕ*_R_(**r**) = 0, with points on the contours separated by the grid size. The direction of each linecut perpendicular to the DW is determined by taking the gradient of the image (∇(*ϕ*_L_(**r**) − *ϕ*_R_(**r**))). Before taking the gradient, the image is first denoised using a Gaussian filter $\exp ((| {\bf{r}}{| }^{2}/(2{\sigma }^{2})))$ with *σ* = 1.5 grid size. Each DW profile along the linecut *M*_*z*_(*x*) is obtained using two-dimensional linear interpolation of the image with *x* ranging between –7 and 7 grid pixels. To further reduce the effect of noise on fitting, we bin DW profiles at every three neighbouring DW positions assuming that the DW properties do not change drastically within ~28-nm distance along the DW. The fitting is conducted with nonlinear least squares method. For small magnetic domains with sizes close to the length of linecut *x*, the wall on the opposite side of the neighbouring domain may affect the magnitude of *M*_*z*_(*x*) at the ends of the linecuts. Therefore, the fitting is weighted such that points with *x* ∈ [−2, 2] pixel (*x* ∈ [−4, 4] grid size) are weighted eight times compared with other points on the linecut.

## Online content

Any methods, additional references, Nature Portfolio reporting summaries, source data, extended data, supplementary information, acknowledgements, peer review information; details of author contributions and competing interests; and statements of data and code availability are available at 10.1038/s41563-026-02583-w.

## Supplementary information

Supplementary information Supplementary Texts 1–5 and Figs. 1–10.

## Data Availability

The data used to produce the figures in this work are available via Edmond at 10.17617/3.XE0QNH. Additional data that support the findings of this study are available from the corresponding authors upon request.
