# Supplementary material for: Sub-wavelength extreme ultraviolet microscopy reveals domain-wall stability during ultrafast demagnetization
Source: Nat Mater. 2026 Apr 20;25(9):1564–71. doi: 10.1038/s41563-026-02583-w (PMC13518233; doi:10.1038/s41563-026-02583-w)
Supplement: Supplementary file 1 [file 41563_2026_2583_MOESM1_ESM.pdf]

nature materials

Supplementary information

https://doi.org/10.1038/s41563-026-02583-w

Sub-wavelength extreme ultraviolet microscopy reveals domain-wall stability during ultrafast demagnetization

In the format provided by the authors and unedited

CONTENTS

S1. Design and optimization of the experimental setup 1

S2. Characterization of spatial resolution 2

S3. The accuracy and precision of extracted domain wall properties 3

S4. Signature of stochastic switching 10

S5. Phenomenological theory of domain wall profiles 13

References 15

S1. DESIGN AND OPTIMIZATION OF THE EXPERIMENTAL SETUP

The design of our experimental setup was guided by the need to optimize the coherence, wavefront, pulse duration, spectral coverage, flux density and long-term stability of the XUV beam. The high-harmonic generation (HHG) scheme ensures high coherence of the emitted XUV light. However, typical XUV beams produced via HHG features a double-Gaussian shape due to contributions from both short and long trajectories in electron recombination [1]. In this work, we generate circularly polarized XUV beams via a trefoil driving field [2]. This HHG scheme encompasses strict selection rules that strongly suppress long–short trajectory degeneracy, yielding harmonics with cleaner wavefront and mode profiles.

To preserve this intrinsic coherence and clean wavefront through the beamline, we use a custom time-preserving monochromator with only one reflective surface, combined with fine alignment control in six degrees of freedom. Wavefront quality is further optimized and verified in situ using a Hartmann sensor [3] and a complimentary double-pinhole coherence measurements [4] ensuring diffraction-limited performance at the sample.

To maintain long-term stability of the system, the pump and probe beam pointing are actively stabilized. In addition, the duration of the driving laser pulse is stabilized by a motorized grating compressor after the Ti:sapphire amplifier with feedback from a 2-photon photodiode sensor. Furthermore, the Ti:sapphire laser is put on a water-cooled base plate to minimize temperature instability. We choose an in-line scheme to produce the trefoil

field for HHG [5]. Here the second harmonic of the 800 nm driving field is first produced with a β-barium borate (BBO) crystal. The co-propagating 400 nm and 800 nm beam are delay-compensated with a set of calcite plates and finally, both beams become circularly polarized with a superachromatic quarter waveplate. In this in-line generation scheme, the 400 nm and 800 nm pulses are intrinsically phase-locked, further enhancing the stability of the system.

To fully exploit the high coherence of the XUV beam for imaging, we enhance the signal at high scattering angles by careful design of the imaging mask. Since magnetic scattering decays rapidly with increasing momentum transfer, it is crucial to enhance the weak high-q signal that ultimately limits the image resolution. To this end, we produce multiple auxiliary holes near the circular field-of-view on the imaging mask (Fig. S7). The interference of the transmitted XUV light from the tightly-spaced auxiliary holes brings the signal at high scattering angles above the noise floor of the camera. Finally, to account for possible reduction of beam coherence due to defects in the beamline, a particular phase-retrieval scheme is applied, which is detailed in Methods.

S2. CHARACTERIZATION OF SPATIAL RESOLUTION

In coherent diffractive imaging, the real-space pixel size is determined by the numerical aperture and wavelength. However, the actual spatial resolution does not necessarily reach this limit. Instead, it is governed by the maximum scattering angle that can be reliably reconstructed, which is the highest diffraction signal recorded with sufficient signal-to-noise ratio (SNR).

After real-space reconstruction, image quality and resolution can be assessed using far-field methods such as the phase retrieval transfer function (PRTF) and Fourier shell correlation (FSC or Fourier ring correlation for 2D data), which evaluate phase consistency and compare correlations from independent measurements. If far-field phases are consistently reconstructed across the entire momentum space, i.e. for all recorded spatial frequencies, the image can be considered diffraction-limited, with the true pixel resolution defined by the momentum transfer at the edge of the detector [6].

A similar approach applies to FSC analysis, where the correlation coefficient is compared to threshold criteria as a function of spatial frequency. The two most commonly used

thresholds are the 0.143 and half-bit criteria [7, 8]. However, far-field estimates should be validated with real-space analysis, such as measuring the smallest resolvable feature or using the knife-edge technique.

Figure S1a shows a typical diffraction pattern recorded with this setup and the mask design (see Fig. S7), optimized for the uniform filling of momentum space. The white dashed line highlights the spatial frequency at the edge of the detector, $q = \frac{1}{2\times(\text{pixel size})} \approx 37\ \mu\text{m}^{-1}$, which determines the real-space pixel size of 13.5 nm. Figure S1b compares the scattered data with the reconstructed signal, showing good agreement between the measured and reconstructed data. Even at spatial frequencies beyond $q = 37\ \mu\text{m}^{-1}$, where the scattering signal is extremely weak, the underlying diffraction fringes are successfully recovered (cf. Fig. S1b). For spatial frequencies below 37 μm^{-1}, the retrieved data are indistinguishable from the measurement. Figure S1c shows the magnetic image from the far-field data in Fig. S1a as reconstructed without interpolation. Here, the magnetic signal within the circular field-of-view is shown as the phase difference between two data sets recorded with opposite helicities. The magnified region highlights multiple domain wall (DW) profiles, which are resolved within a single pixel transition, demonstrating isotropic true-pixel resolution in real space. Far-field analysis is shown in Fig. S1d, where the PRTF value computed for the single helicity image (Fig. S1a) remains above 0.75 across the momentum space. This confirms that all spatial frequencies are consistently reconstructed irrespective of the initial guess. Additionally, several FSC plots, computed from different types of image contrast for (i) the entire exit-surface field including the signal from auxiliary holes, and (ii) isolated circular FOV images, also support a diffraction-limited resolution of 13.5 nm irrespective of the image contrast mechanism used.

S3. THE ACCURACY AND PRECISION OF EXTRACTED DOMAIN WALL PROPERTIES

Results with different numerical apertures. To characterize the accuracy of the measured DW width and its potential overestimation due to the inherent point spread function of the imaging system, we performed a set of measurements on the same sample using a reduced numerical aperture (NA) that limits spatial resolution to 18.9 nm (cf. Fig. S2, note that this is the same sample shown in Fig. S1, now shown in inverted colormap). The average

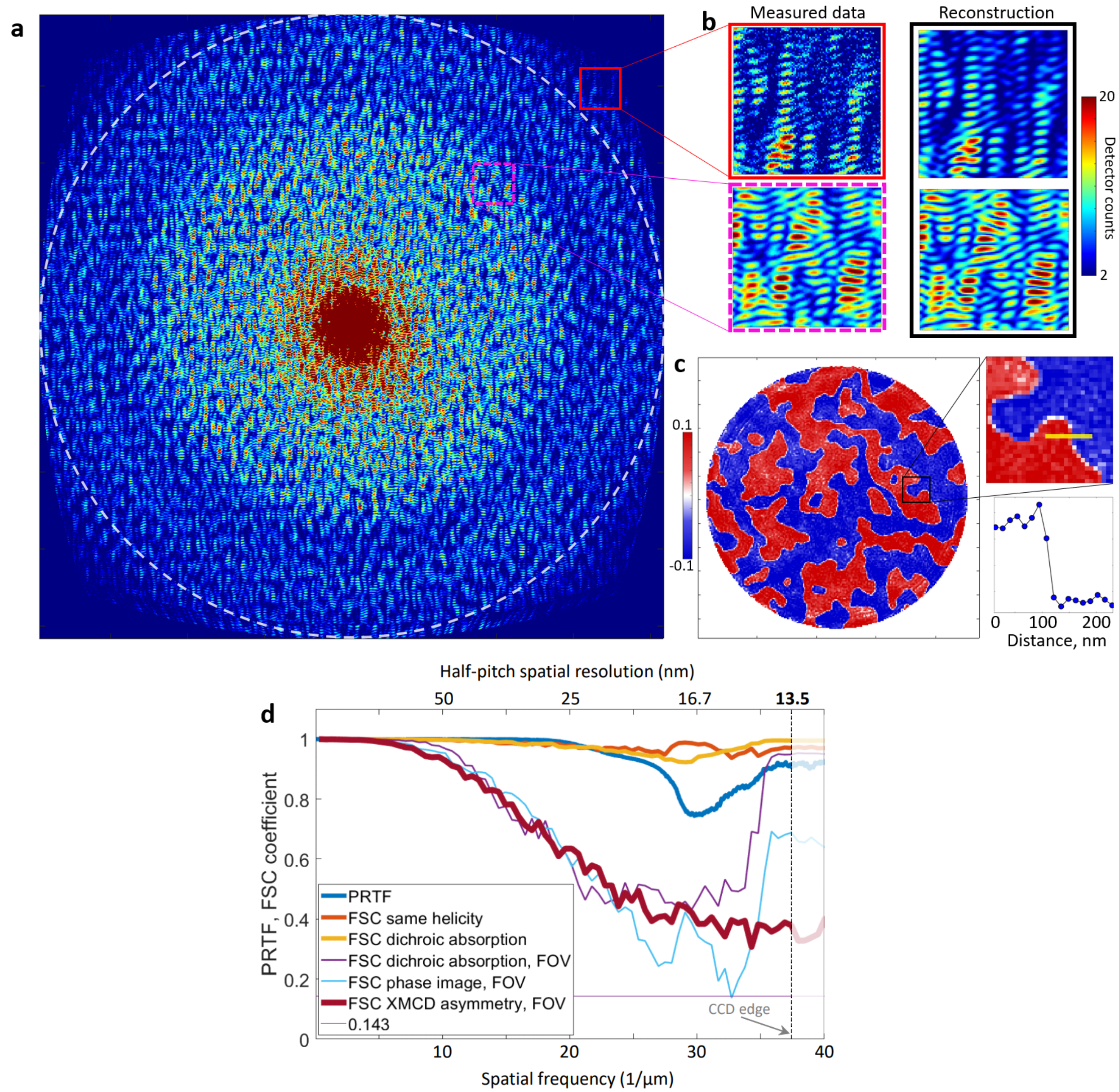

FIG. S1. Spatial resolution verification. **a** Far-field scattering data recorded with a 10-minute exposure time. The dashed line indicates the spatial frequency at the edge of the CCD (37 μm^{-1}, defining a real-space pixel size of 13.5 nm. **b** Comparison of the measured data with the reconstructed far-field shows good agreement, even in the corners of the detector, where the SNR is weakest. **c** Corresponding reconstruction of the isolated magnetic signal shown as reconstructed in true-pixel without interpolation. The zoom-in regions highlights where DWs are resolved within a single pixel transition, indicating true-pixel resolution in real-space. **d** Far-field analysis using the phase retrieval transfer function (PRTF) and Fourier shell correlations (FSC) demonstrates reliable imaging across all recorded spatial frequencies and different types of image contrast.

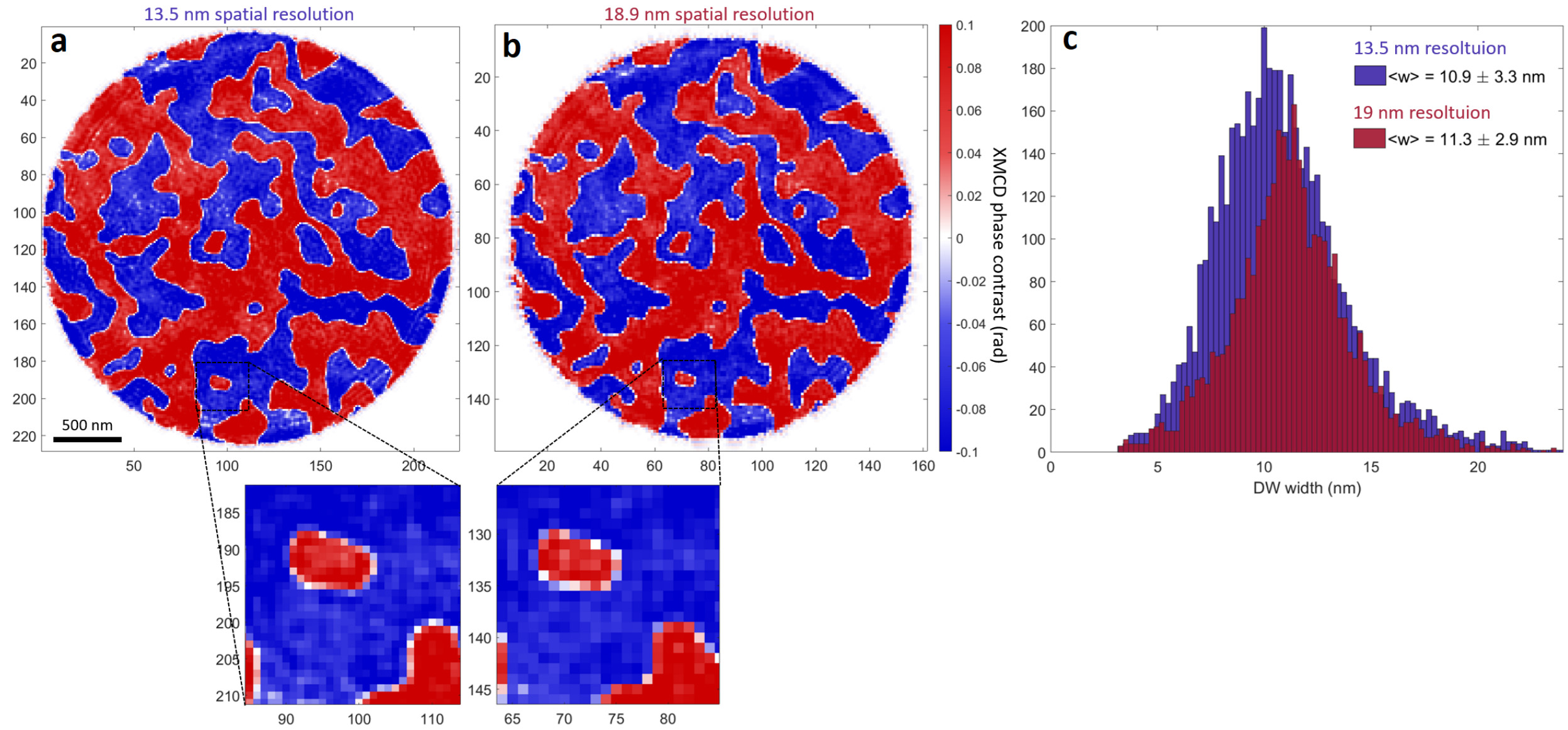

FIG. S2. Comparison of domain wall measurement analysis for the data recorded at relaxed imaging resolution. **a** Magnetic state recorded with 13.5 nm spatial resolution using XMCD phase contrast imaging. **b** Same sample imaged with 18.9 nm spatial resolution. **c** Local DW width analysis results for both images. The relative change in resolution is substantially larger than the relative change in the measured mean values $\bar{w}$, which remains within the uncertainty of the absolute DW width between independent datasets recorded at different NA (see text). The relative change in the resolution used is substantially larger than the relative change of the measured mean values $\bar{w}$ which is within the uncertainty of the absolute value of DW width between independent sets (see text).

DW width measured in several thousand linecuts across the FOV is 10.9 nm, with a standard deviation of 3.3 nm when the sample is imaged with 13.5 nm spatial resolution. For the dataset recorded with reduced NA, the mean value $\bar{w}$ increases to 11.3 nm, which is significantly lower than the difference in resolution. Since higher resolution imaging requires substantially longer exposure time to ensure sufficient SNR for high spatial frequencies, all time-resolved data shown in, Figs. 2-4 of the main text were recorded at the reduced spatial resolution of 18.9 nm, while still maintaining sufficient precision for local domain wall width measurements.

Effect of noise. The precision of the extracted DW width and position is strongly influenced by the SNR of the image. High-frequency noise (see the typical linecut in Fig. S1c)

primarily originates from the noise in the diffraction data at the highest scattering angles (cf. Fig. S1b). This can be partially suppressed by applying a super-Gaussian filter to the edges of the far-field data before real-space analysis. The filter consists of a circular, quasi-binary mask with diameter matching the size of the detector. The value of the mask inside the circle is 1 and outside 0.3. To de-noise the signal, the mask is first smoothed by a super-Gaussian filter

$$G(\mathbf{k}) = \left(e^{-\frac{k^2}{2\sigma_g^2}} \right)^P ,$$

with $\sigma_g = 15$ pixels and power $P = 3$. The final image is obtained by multiplying the reciprocal-space image of the final reconstruction with the filter and performing an inverse Fourier transform. The application of the filter reduces $\sigma_{\delta w}$ (see Fig. 2c in the main text) by up to 2 nm while increasing the mean value of DW widths $\bar{w}$ by 2–4%. As we are mainly interested in the *relative* changes of DW properties in time-dependent measurements, such slight increase in overall DW width does not affect our conclusions.

To further examine the impact of noise on extracted DW properties, we perform Monte-Carlo calculations with simulated DW profiles. First, we extract the noise level of the measured real-space images by fitting a Gaussian to the histogram of absolute magnetic contrast $|\phi_L - \phi_R|$ (Figs. S3a,b). Second, a DW profile is generated with Eq. (1) in the main text with artificially set w and M_0 according to fitting results from unpumped data. Here, the position x_0 is set to 0. Third, Gaussian noise with the noise level from the first step is added to the profile, and finally, we perform a fitting with Eq. (1) in the main text to see the deviation of fitting parameters from the original value (Fig. S3c). To account for partially demagnetized conditions, M_0 is modified to reflect the level of demagnetization. The width w for the generated profiles are set to 15 nm, in accordance with empirical value. For each M_0, we generate 10000 DW profiles with the aforementioned procedure and obtain the distribution of fitted width (w_{MC}) and position ($x_{0,\mathrm{MC}}$). Note that the result of the Gaussian fitting to the histogram of $|\phi_L - \phi_R|$ contains contribution from both experimental noise and intrinsic variations in magnetization due to sample inhomogeneity. Such potential overestimation of noise level will cause the standard deviations of the distributions of w_{MC} and $x_{0,\mathrm{MC}}$ to be larger than those from experimental data. Figure S5 shows a comparison of the empirical distributions of $|x_0 - x_0^{\mathrm{ref}}|$ (cf. Fig. 3d in main text) and the results from Monte-Carlo calculations with 10000 samples. The good agreement between experimental

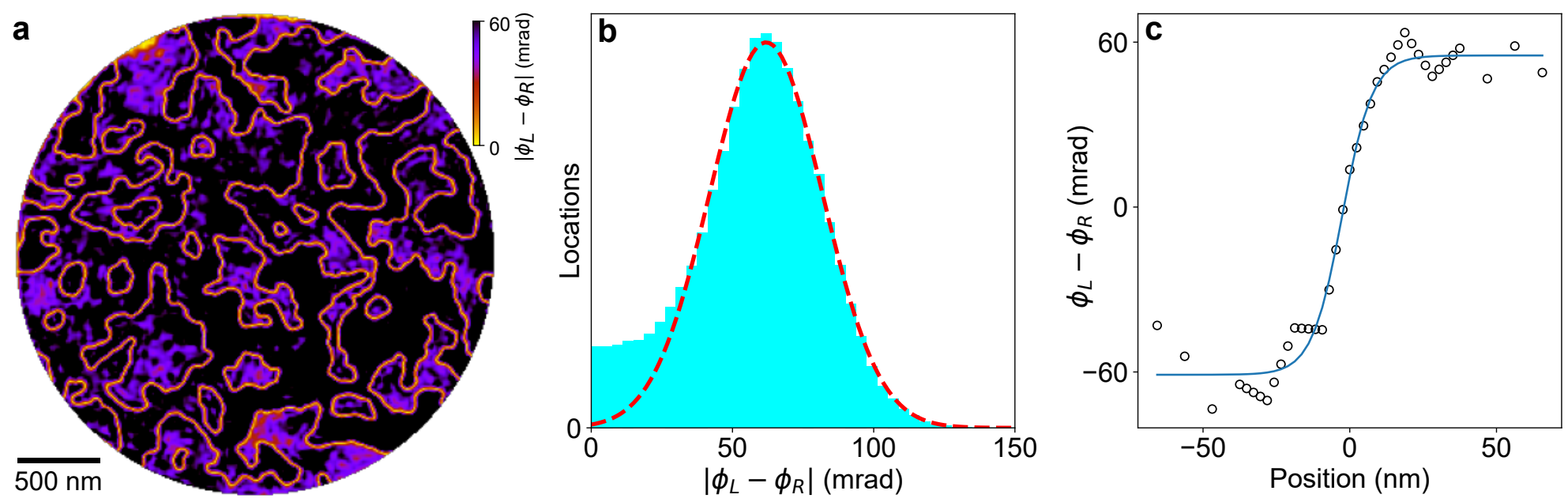

FIG. S3. Extraction of noise level from acquired images for Monte-Carlo simulations. **a** Absolute value of phase contrast of a reconstructed magnetic image in unpumped state. **b** Extraction of noise level by fitting a Gaussian (red dashed line) to the histogram of absolute phase contrast in cyan. **c** An example of simulated DW profile (black circles) and its fit with Eq. (1) in main text (blue line).

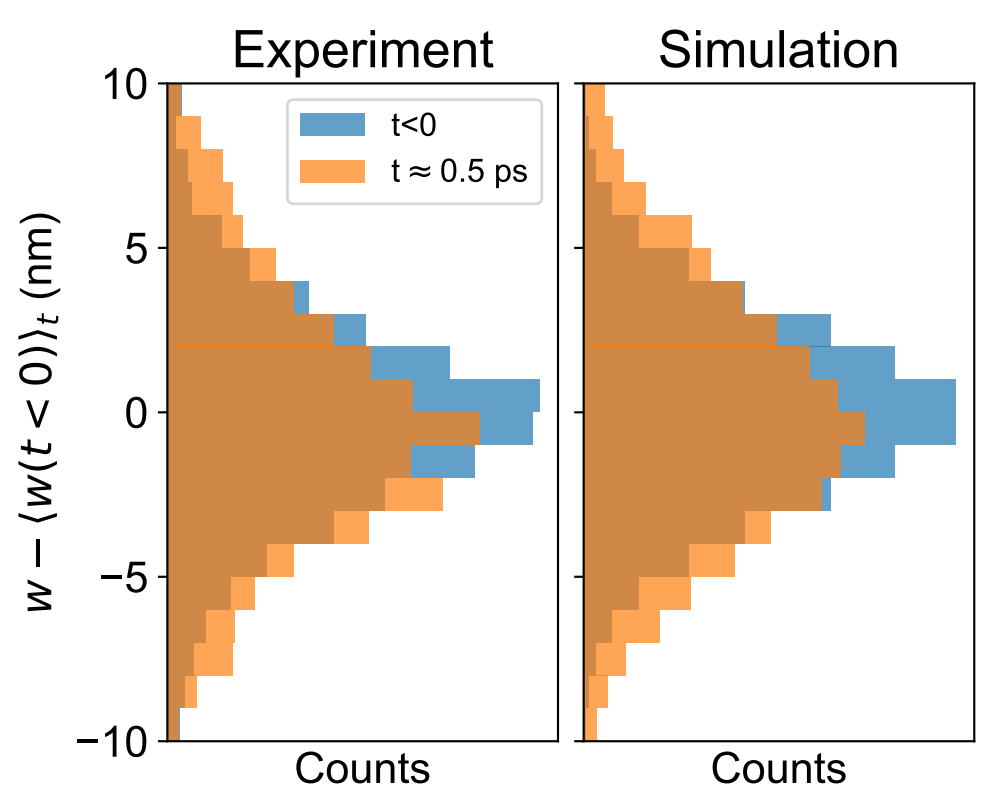

FIG. S4. Increase of uncertainty in fitted DW width due to changes of signal-to-noise ratio (SNR). This figure shows a comparison of the empirical changes in width distribution (left panel, cf. Fig. 3c in main text) and the distribution from Monte Carlo simulation with different SNR. Here the ratio between the SNR in the simulations for the two histograms in the right equals the ratio of M_0 for the two time delays (left panel).

results and simulation confirms that the distribution of $|x_0 - x_0^{\mathrm{ref}}|$ is mainly contributed by noise.

Resolving local domain wall instability. Although the distributions of DW width (w_{avg}, w) and displacement from experimental data stipulate the overall changes in DW properties, it may not be straightforward in reflecting rare events such as DW displacements that only

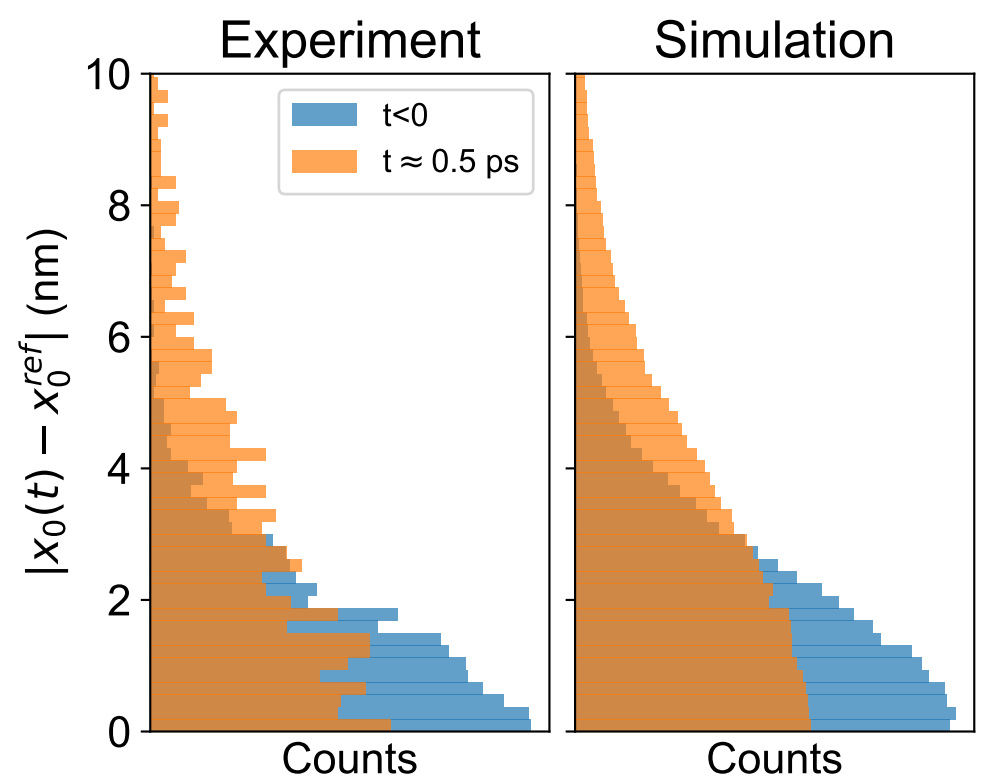

FIG. S5. Increase of the DW localization uncertainty for partially demagnetized sample due to a reduction of XMCD contrast. The left panel shows the magnitude of DW displacement (cf. Fig. 3d in main text) and the right panel shows the corresponding results from Monte Carlo simulations with the ratio of SNR equal to the ratio of M_0 in experimental data (left panel).

occur at specific locations, e.g. where the DW contour has a large curvature [9]. In such cases, the derived 0.2 nm precision for the average DW width, for example, may be insufficient to resolve displacements as large as 10 nm if they occur at only a small percentage of DW positions. To accurately assess such DW motions, we focus on local measurements. In addition to the application of the super-Gaussian filter and binning neighboring DW profiles as described in Methods, we further compare results from averaged images from negative delays, delays between 0.1 ps and 1.8 ps (maximal demagnetization), and 2.3 ps-6.3 ps (recovery), with results from individual delays to recover possible displacements at a small number of locations from noise. In Figure S6, the absolute values of the displacement for averaged images at maximal demagnetization and recovery are plotted with a blue-green colormap in Figs. S6a,b and the corresponding histograms are shown in Fig. S6c. In both cases, the standard deviations of the distributions are 1.6 nm, which corresponds to the maximally possible root-mean-square displacement. Note that the value of 1.6 nm is nearly identical to the average of $|x_0 - x_0^{\mathrm{ref}}|$ at negative delays (see main text Fig. 3d). In addition, no correlation is observed between the local curvature of the domain wall and the extracted displacement value (Figs. S6a,b). The analysis here further confirms that the extracted changes of DW positions are mainly due to noise, and the upper limit of 3 nm displacement provided in the main text is a conservative estimation.

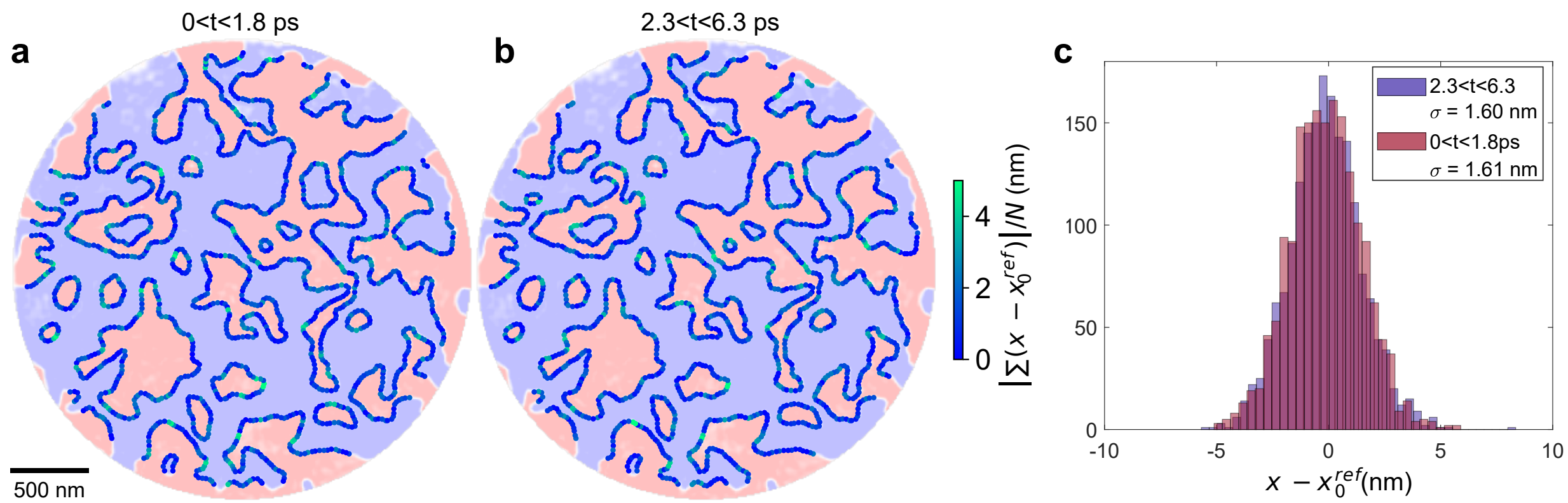

FIG. S6. Domain wall position stability. **a** Computed domain wall displacement map for the DW locations identified at the average image of the magnetic state between 0 and 1.8 ps vs positions at the reference point at negative delays. **b** Local displacement relative to the negative delay, computed for the averaged image of delays between 2.3-6.3 ps. The background is the reference image taken at negative delays. Dot colors correspond to the magnitude of the local DW displacement of the DW center compared to the positions at a negative delay, x_0^{ref}. The corresponding histograms are shown in **c** for both histograms.

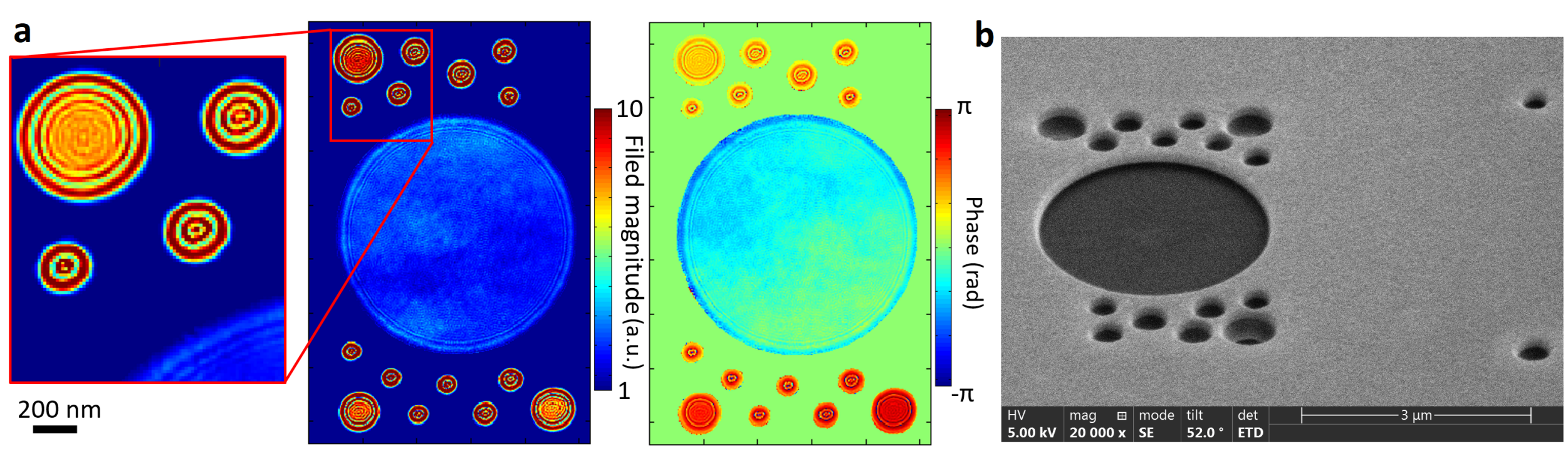

FIG. S7. **a** Reconstructed magnitude and phase of the scattered wave after illumination with left-circularly polarized light. The magnified region highlighting the beating of guided modes within the auxiliary holes and at the edge of the FOV aperture. **b** Tilted view of the mask design captured with scanning electron microscope. The size and arrangement of the auxiliary holes are optimized to ensure a homogeneous far-field diffraction pattern with high scattering angles.

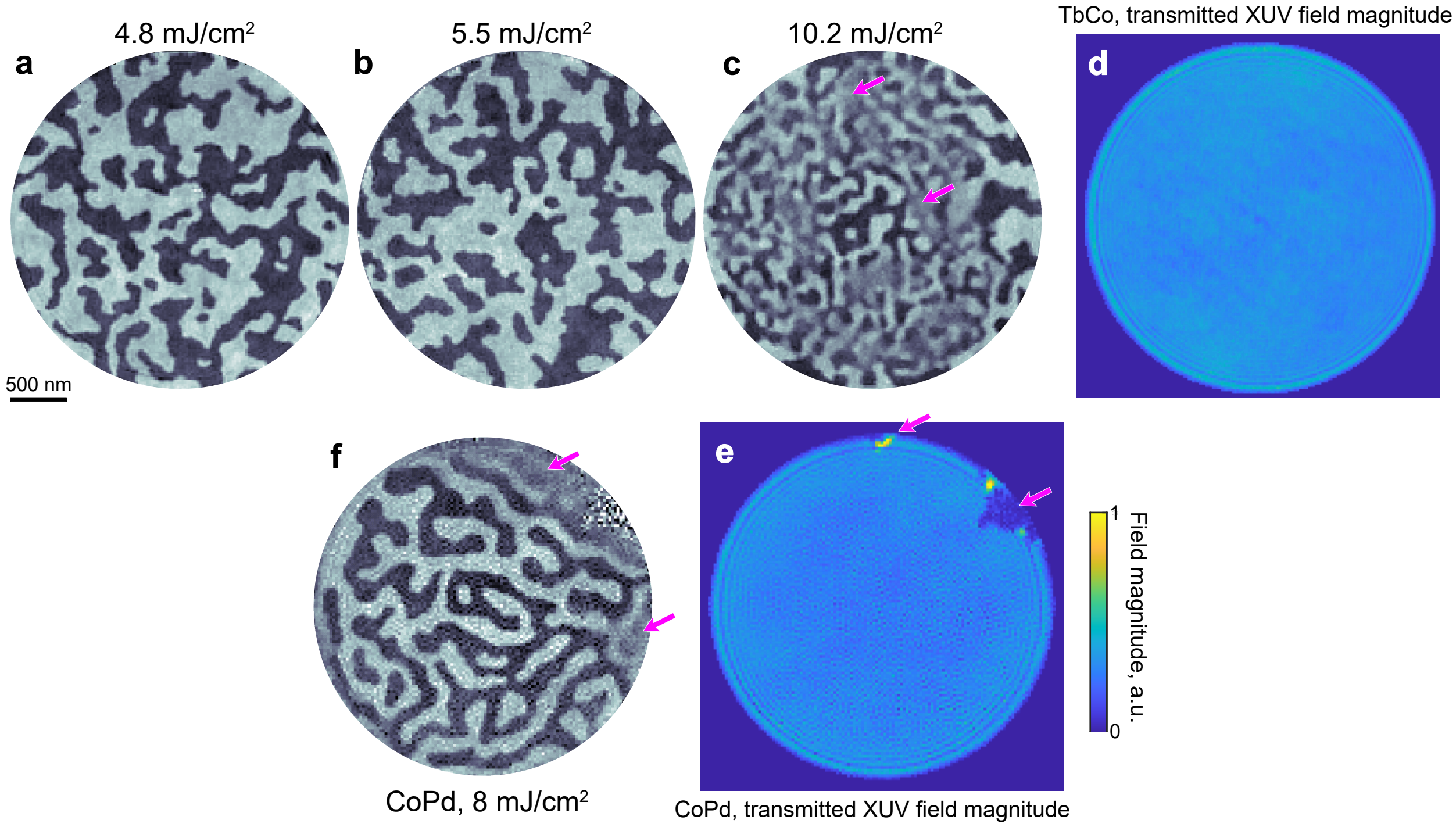

FIG. S8. **a–c** Magnetic domain patterns of the TbCo sample recorded at negative delays ($t < 0$) at different FOV locations and pump fluences. **d** Transmitted field amplitude of the XUV probe beam through the FOV shown in **c**. **e,f** Transmitted field amplitude of the CoPd multilayer sample (e) and the corresponding snapshot of the magnetic domain pattern in this FOV (f). The blurred regions with reduced magnetic contrast, marked by magenta arrows, result from irreversible changes in the domain pattern during the exposure time reflecting incoherent averaging over different magnetic configurations occurring between successive pump–probe cycles in the stroboscopic experiment. Regions with distinct XUV transmission compared to the neighboring area, indicated by magenta arrows in (e), correspond to areas where the magnetic film was damaged by the pump laser.

S4. SIGNATURE OF STOCHASTIC SWITCHING

In a stroboscopic imaging experiment, the acquired signal on the camera for each individual image is the incoherent sum of the transmission through the sample over multiple pump-probe cycles at one specific time delay. Denoting the transmitted intensity of the kth cycle as I_k, the camera signal

$$I_{tot} = \sum_{k=1}^{N} I_k.$$

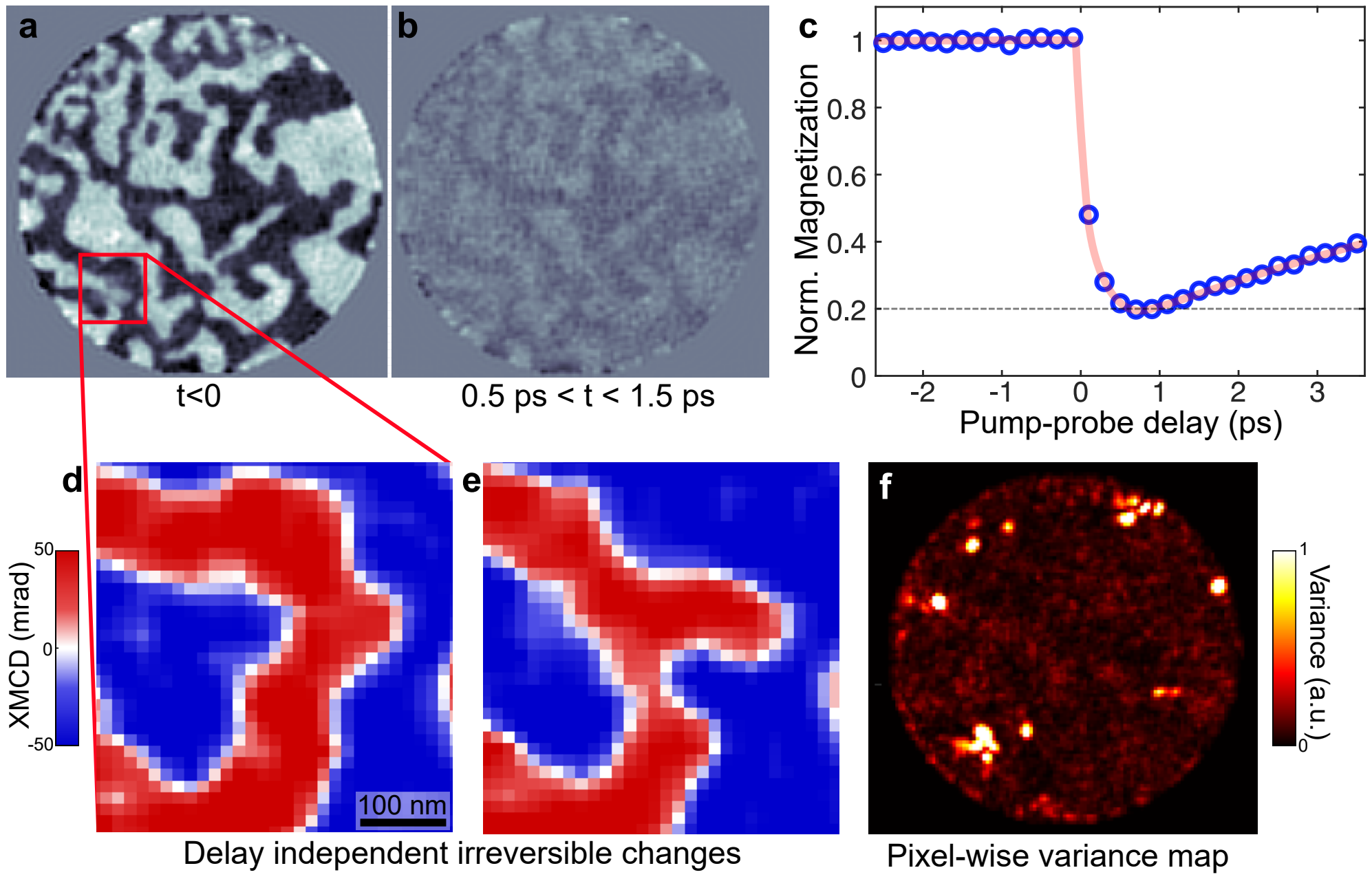

FIG. S9. Spin dynamics in $Tb_{25}Co_{75}$ sample at high pump fluence recorded with short exposure time and 28 nm spatial resolution. **a** Image at a negative pump-probe delay (probe before excitation). Some regions show reduced contrast due to averaging over stochastic irreversible changes during the exposure. **b** Image obtained by averaging frames between 0.5 and 1.5 ps delay, corresponding to maximum magnetization suppression (80%). Several frames were combined to improve SNR. **c** Transient magnetization curve, extracted from regions remained reversible throughout the sequence. **d-e** Zoom into representative negative-delay frames recorded with short exposure time, illustrating local stochastic domain rearrangements between individual acquisitions. The relaxed spatial resolution in this data set enabled shorter exposure, allowing some individual switching events between acquisitions to be captured. **f** Pixel-wise variance map of negative-delay images, highlighting delay-independent irreversible changes (bright spots), as also visible in n **d,e**.

If the pump-induced changes are reversible, $I_1 = I_2 = \ldots = I_N = I$ and $I_{tot} = NI$. On the other hand, if the sample switched between different states during the acquisition period, the camera signal I_{tot} can be written as

$$I_{tot} = \sum_p n_p I_p, \tag{1}$$

with $\sum_p n_p = N$. Here the index p represents the state of the sample and n_p the number of cycles where the sample is in state p.

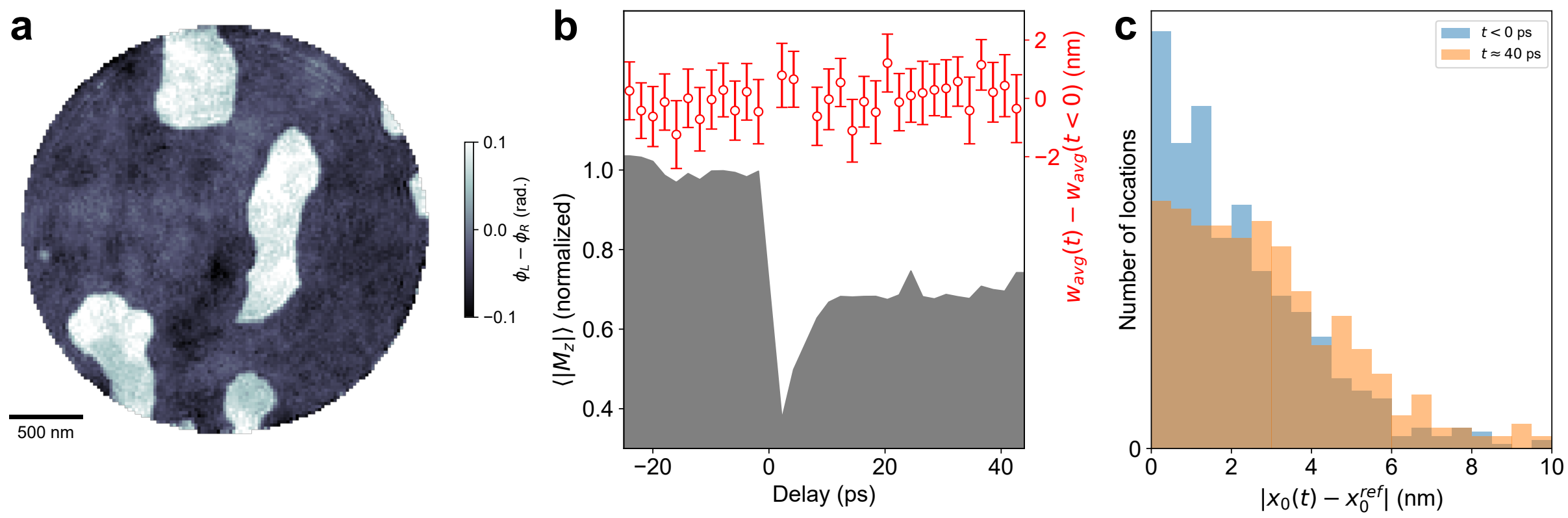

FIG. S10. DW widths and displacements measured up to 40 ps time delay. **a** Static image of the sample. **b** Change in magnetization (gray shade) and widths of spatially averaged DW profiles (w_{avg}, red circles). The maximal uncertainty of fitting the spatially averaged DW profiles is 1.2 nm (error bar) and the distribution of $w_{avg}(t)$ falls within a range (1σ) of 0.6 nm. The number of position points for a DW profile is described in Methods. **c** Histogram of local DW displacement at negative delays (blue) and around 40 ps (orange). Average displacement $\langle|x_0(t) - x_0^{ref}|\rangle$ at -13 ps (blue) and 40 ps (orange) are 2.2 nm and 3.0 nm, respectively.

To extract the sample magnetization in real space, the transmitted electric fields are reconstructed from the acquired intensity I_{tot}. In our experiment, the transmitted fields can be separated into two components: ones through the reference holes E_{ref} and those through the field of view E_{FOV}. The transmitted intensity for each pump-probe cycle I_k is thus $|E_{\mathrm{ref}} + E_{\mathrm{FOV},k}|^2$. The index k is dropped for the reference field term as it should be independent of the pump-probe cycles. For reversible dynamics, the acquired signal I_{tot} is linked to the fields as $I_{tot} = N|E_{\mathrm{ref}} + E_{\mathrm{FOV}}|^2$ and E_{FOV} can be directly reconstructed. However, when the camera signal is an incoherent sum from multiple different states, the reconstruction is no longer straightforward [10]. To overcome this problem, the imaging mask is designed such that the dominant contribution to the transmitted field is from the reference holes (cf. Fig. S7a), i.e. $|E_{\mathrm{ref}}| \gg |E_{\mathrm{FOV}}|$. Here, the transmission I_p can be approximated as

$$I_p = |E_{\mathrm{ref}} + E_{\mathrm{FOV},p}|^2 = |E_{\mathrm{ref}}|^2 + 2E_{\mathrm{ref}}E_{\mathrm{FOV},p} + |E_{\mathrm{FOV},p}|^2 \approx |E_{\mathrm{ref}}|^2 + 2E_{\mathrm{ref}}E_{\mathrm{FOV},p}. \quad (2)$$

Now, Eq. (1) can be expressed as

$$I_{tot} = N|E_{\mathrm{ref}}|^2 + 2E_{\mathrm{ref}} \sum_p n_p E_{\mathrm{FOV},p}. \tag{3}$$

In this case, the real-space reconstruction over I_{tot} yields a weight-averaged field $E_{\mathrm{avg}} = \sum_p n_p E_{\mathrm{FOV},p}$ in the FOV region. Considering the pump-induced changes, we rewrite the field E_{avg} as

$$E_{\mathrm{avg}} = E_{\mathrm{pumpoff}} \cdot \sum_p n_p |\Delta E_p| e^{i\Delta\phi_p}/N; \tag{4}$$

the term $|\Delta E|$ represents the pump-induced change in the transmitted field amplitude and $\Delta\phi$ the phase. Because the magnetization is proportional to the phase of the field [11] and the pump-induced changes are small, viz. $|\Delta E|$ is close to 1 and $\Delta\phi$ to zero, the vector sum $\sum_p n_p|\Delta E_p|e^{i\Delta\phi_p}/N$ over the complex plane results in an average phase $\Delta\phi_{\mathrm{avg}} = \sum_p n_p \Delta\phi_p/N$. Therefore, the extracted phase $(\phi_L - \phi_R)$ for a stochastically switching sample represents the weighted average of the sample's magnetic states (cf. Figs. S8c,f). As switching involves changing between positive and negative values of phase $(\phi_L - \phi_R)$, the magnitude of the reconstructed phase $|(\phi_L - \phi_R)|$ for regions undergoing switching during the data acquisition is reduced.

S5. PHENOMENOLOGICAL THEORY OF DOMAIN WALL PROFILES

Within continuum theory, the domain wall width w is described based on the effective magnetic anisotropy K and exchange stiffness A:

$$w = \sqrt{\frac{A}{K}}. \tag{5}$$

In equilibrium, w is temperature dependent due to the magnetization dependence of the micromagnetic parameters. In particular, the seminal theory of Callen et al [12], shows that the temperature dependence of the uniaxial anisotropy of a cubic ferromagnet can be expressed as

$$K(T) = K_0\, m(T)^3 \tag{6}$$

where $m(T) = M_s(T)/M_s(0)$ is the reduced magnetization and $M_s(T)$ the saturation magnetization at temperature T. Also the exchange stiffness exhibits a temperature dependence.

In a mean-field picture, the Landau theory of phase transitions predicts a linear dependence of the exchange stiffness, consistent with a magnetization dependence as follows:

$$A(T) = A_0\, m(T)^2. \tag{7}$$

These phenomenological expressions show that domain walls broaden under the increase of temperature $\delta(T)/\delta_0 = m(T)^{-1/2}$. The physics of these temperature dependencies of the micromagnetic parameters can be understood from a coarse graining approach. Assuming temperature-independent single-ion anisotropies and exchange interactions between atomistic spins, effective anisotropies and exchange stiffness for an ensemble of spins are obtained by expanding the free energy for small deviations from the perfectly aligned FM ground state, yielding thermal expectation values $\langle \cos^2\theta \rangle$, where $\theta = 0$ corresponds to the FM state. Likewise for exchange stiffness, the temperature dependence follows from $\langle \cos(\theta_{ij}) \rangle$, θ_{ij} the relative angle between two neighboring spins.

Numerical calculations confirm the validity of the scaling relations, but generally, different exponents are obtained. In the present case, the thin-film limit is of importance, since for this case, a nearly uniform heating of the system across the film thickness is present, and a non-equilibrium relation between reduced magnetization and micromagnetic parameters may be expected. In this case, the single-ion anisotropy does not provide an adequate description, and an ion-ion anisotropic exchange interaction gives the leading contribution [13–15]. This leads to a scaling exponent that is much closer to that of the exchange stiffness:

$$K(T) = K_0 m(T)^{2+\kappa}, \tag{8}$$

where $0 < \kappa \ll 1$. For the exchange stiffness, corrections beyond the mean-field take the form Ref. [16]:

$$A(T) = A_0 m(T)^{2-\varepsilon}, \tag{9}$$

where $0 < \varepsilon \ll 1$.

Under femtosecond laser excitation, $m(t)$ reduces faster than the magnetic system can reach internal equilibrium. Hence, being coarse-grained parameters, the fastest changes to the domain wall widths are expected when the micromagnetic parameters change in step with the magnetization:

$$w[m(t)]/w_0 = m(t)^{-(\kappa+\varepsilon)/2}. \tag{10}$$

Taking literature values $\kappa = 0.1$ and $\varepsilon = 0.24$, reported for FePt [13, 16], we obtain $w[m(t)]/w_0 = 1.125$ for a maximum reduction of $m(t)$ by 50%. Hence, the maximum estimated domain wall broadening for a 15 nm wall is 1.9 nm, consistent with the experimental error bar. We emphasize that this estimation gives only an upper bound, on short time scales the possible changes are smaller. In particular, on time-scales shorter than $t \ll w_0/v_{\mathrm{g}} \sim 100$ps, with $v_{\mathrm{g}} \sim 0.1$ nm/ps the highest magnon group velocity, domain wall profiles cannot follow the magnetization instantaneously.

---

[1] P. Balcou, P. Sali'eres, A. L'Huillier, and M. Lewenstein, Physical Review A **55**, 3204 (1997).

[2] O. Kfir, P. Grychtol, E. Turgut, R. Knut, D. Zusin, D. Popmintchev, T. Popmintchev, H. Nembach, J. M. Shaw, A. Fleischer, H. Kapteyn, M. Murnane, and O. Cohen, Nature Photonics **9**, 99 (2015).

[3] T. Mey, S. Zayko, C. Ropers, B. Schäfer, and K. Mann, Opt. Express **23**, 15310 (2015).

[4] C. Chang, *Coherence Techniques at Extreme Ultraviolet Wavelengths*, Ph.D. thesis, University of California, Berkeley (2002).

[5] O. Kfir, E. Bordo, G. Ilan Haham, O. Lahav, A. Fleischer, and O. Cohen, Applied Physics Letters **108**, 211106 (2016).

[6] H. N. Chapman, A. Barty, S. Marchesini, A. Noy, S. P. Hau-Riege, C. Cui, M. R. Howells, R. Rosen, H. He, J. C. H. Spence, U. Weierstall, T. Beetz, C. Jacobsen, and D. Shapiro, Journal of the Optical Society of America A **23**, 1179 (2006).

[7] P. B. Rosenthal and R. Henderson, Journal of Molecular Biology **333**, 721 (2003).

[8] M. van Heel and M. Schatz, Journal of Structural Biology **151**, 250 (2005).

[9] R. Jangid, N. Z. Hagström, M. Madhavi, K. Rockwell, J. M. Shaw, J. A. Brock, M. Pancaldi, D. De Angelis, F. Capotondi, E. Pedersoli, H. T. Nembach, M. W. Keller, S. Bonetti, E. E. Fullerton, E. Iacocca, R. Kukreja, and T. J. Silva, Physical Review Letters **131**, 256702 (2023).

[10] C. Klose, F. Büttner, W. Hu, C. Mazzoli, K. Litzius, R. Battistelli, S. Zayko, I. Lemesh, J. M. Bartell, M. Huang, C. M. Günther, M. Schneider, A. Barbour, S. B. Wilkins, G. S. D. Beach, S. Eisebitt, and B. Pfau, Nature **614**, 256 (2023).

[11] O. Kfir, S. Zayko, C. Nolte, M. Sivis, M. Möller, B. Hebler, S. S. P. K. Arekapudi, D. Steil, S. Schäfer, M. Albrecht, O. Cohen, S. Mathias, and C. Ropers, Science Advances **3**, eaao4641

(2017).

[12] H. b. Callen and E. Callen, Journal Physical Chemistry Solids **27**, 1271 (1966).

[13] J.-U. Thiele, K. R. Coffey, M. F. Toney, J. A. Hedstrom, and A. J. Kellock, Journal of Applied Physics **91**, 6595 (2002).

[14] J. B. Staunton, S. Ostanin, S. S. A. Razee, B. L. Gyorffy, L. Szunyogh, B. Ginatempo, and E. Bruno, Physical Review Letters **93**, 257204 (2004).

[15] P. Asselin, R. F. L. Evans, J. Barker, R. W. Chantrell, R. Yanes, O. Chubykalo-Fesenko, D. Hinzke, and U. Nowak, Physical Review B **82**, 054415 (2010).

[16] U. Atxitia, D. Hinzke, O. Chubykalo-Fesenko, U. Nowak, H. Kachkachi, O. N. Mryasov, R. F. Evans, and R. W. Chantrell, Physical Review B **82**, 134440 (2010).
